# Supplementary material for: Impact of climate and land use on the temporal variability of sand fly density in Sri Lanka: A 2-year longitudinal study
Source: PLoS Negl Trop Dis. 2024 Nov 21;18(11):e0012675. doi: 10.1371/journal.pntd.0012675 (PMC11620634; doi:10.1371/journal.pntd.0012675)
Supplement: S1 Text — Section 1: Sand fly collection. Fig A. Sand fly trap types. Fig B. Placement of the light traps and cattle bated traps. Section 2: Exploratory data analysis. Fig C. Spatial variability and seasonality of sand fly counts. Table A. Summary statistics of monthly sand fly density by sentinel site. Fig D. Box plots showing the seasonality of climate variables averaged across all surveillance settings from March 2018 to February 2020. Fig E. Correlation between each climatic covariate averaged across all nine monitoring stations from March 2018 to February 2020 in Sri Lanka. Table B. Distribution of non-climate variables among surveillance sites. Section 3: Statistical analytical approach. Box A: Statistical Analytical Approach. Section 4: Distributed Lag Nonlinear Models (DLNM). Table C. Sum of the Q-AIC values obtained by the first stage models for LT per trap, CBNT per trap and LT monthly total for each weather variable evaluated. Table D. Definitions of the cross-basis matrix for the selected first stage models. Fig F. Relative risk (RR) of leishmaniasis vector activity (measured by LT) by rainfall at a lag of 0 to 3 months. Fig G. Relative risk (RR) of leishmaniasis vector activity (measured by LT per trap) by ambient temperature (maximum temperature) at a lag of 0 to 3 months. Fig H. Relative risk (RR) of leishmaniasis vector activity (measured by LT per trap) by average relative humidity at a lag of 0 to 3 months. Fig I. Relative risk (RR) of sand fly vector density (measured by LT) by sunshine hours at lag of 0 to 3 months. Fig J. Relative risk (RR) of sand fly vector density (measured by LT per trap) by wind speed at a lag of 0 to 3 months. Fig K. Relative risk (RR) of sand fly vector density (measured by LT per trap) by soil temperature measured at 10 cm from the surface in morning hours (8:30 am) at a lag of 0 to 3 months. Fig L. Relative risk (RR) of sand fly vector density (measured by LT per trap) by evaporation values at a lag of 0 to 3 months. Table E. Qua [file pntd.0012675.s001.docx]

Supporting Information

Table of Contents

[Section 1: Sand fly collection 2](#_Toc182332959)

[Trap types 2](#_Toc182332960)

[**Fig A in S1 Text.** Sand fly trap types 2](#_Toc182332961)

[The Sampling Locations 3](#_Toc182332962)

[**Fig B in S1 Text.** Placement of the light traps and cattle bated traps. 3](#_Toc182332963)

[Section 2: Exploratory data analysis 4](#_Toc182332964)

[**Fig C in S1 Text.** Spatial variability and seasonality of sand fly counts. 4](#_Toc182332965)

[**Table A in S1 Text.** Summary statistics of monthly sand fly density by sentinel site. 4](#_Toc182332966)

[Climate variables 5](#_Toc182332967)

[**Fig D in S1 Text.** Box plots showing the seasonality of climate variables averaged across all surveillance settings from March 2018 to Feb 2020. 5](#_Toc182332968)

[Correlation between climate variables 6](#_Toc182332969)

[**Fig E in S1 Text.** Correlation between each climatic covariates averaged across all nine monitoring stations from March 2018 to February 2020 in Sri Lanka. 6](#_Toc182332970)

[Non-climate land use variables 6](#_Toc182332971)

[**Table B in S1 Text.** Distribution of non-climate variables among surveillance sites. 6](#_Toc182332972)

[Section 3: Statistical analytical approach 7](#_Toc182332973)

[Methods at a glance 7](#_Toc182332974)

[**Box A in S1 Text:** Statistical Analytical Approach 7](#_Toc182332975)

[Section 4: Distributed Lag Nonlinear Models (DLNM) 8](#_Toc182332976)

[**Table C in S1 Text.** Sum of the Q-AIC values obtained by the first stage models for LT per trap, CBNT per trap and LT monthly total for each weather variable evaluated. 8](#_Toc182332977)

[**Table D in S1 Text**. Definitions of the cross-basis matrix for the selected first stage models. 8](#_Toc182332978)

[Full Spectrum of Exposure-Lag-Response Associations 9](#_Toc182332979)

[**Fig F in S1 Text.** Relative risk (RR) of leishmaniasis vector activity (measured by LT) by rainfall at a lag of 0 to 3 months 9](#_Toc182332980)

[**Fig G in S1 Text.** Relative risk (RR) of leishmaniasis vector activity (measured by LT per trap) by ambient temperature (maximum temperature) at a lag of 0 to 3 months. 9](#_Toc182332981)

[**Fig H in S1 Text.** Relative risk (RR) of leishmaniasis vector activity (measured by LT per trap) by average relative humidity at a lag of 0 to 3 months. 10](#_Toc182332982)

[**Fig I in S1 Text.** Relative risk (RR) of sand fly vector density (measured by LT) by sunshine hours at lag of 0 to 3 months. 10](#_Toc182332983)

[**Fig J in S1 Text.** Relative risk (RR) of sand fly vector density (measured by LT per trap) by wind speed at a lag of 0 to 3 months. 11](#_Toc182332984)

[**Fig K in S1 Text.** Relative risk (RR) of sand fly vector density (measured by LT per trap) by soil temperature measured at 10cm form the surface in morning hours (8.30am) at a lag of 0 to 3 months. 11](#_Toc182332985)

[**Fig L in S1 Text.** Relative risk (RR) of sand fly vector density (measured by LT per trap) by evaporation values at a lag of 0 to 3 months. 12](#_Toc182332986)

[**Table E in S1 Text.** Quantification of divisional heterogeneity of the association between weather variables and the LT per trap index obtained by the second stage multi-variate meta-analysis. 12](#_Toc182332987)

[Exposure-lag-response association between weather variables and sand fly density measured by cattle baited net traps (CBNT) 13](#_Toc182332988)

[**Fig M in S1 Text.** Weather and sand fly density measured by CBNT. 13](#_Toc182332989)

[Section 5: Moderator effect of climate zones on weather-Sand Fly association 14](#_Toc182332990)

[**Table F in S1 Text:** Wald test statistics for the moderator effect of climate zones on weather-sand fly association. 14](#_Toc182332991)

[**Fig N in S1 Text.** Moderator effect of climate on weather and Sand Fly density. 15](#_Toc182332992)

[Section 6: Machine learning and XAI 16](#_Toc182332993)

[XGBoost model building and validation 16](#_Toc182332994)

[**Table G in S1 Text.** Stepwise summary of XGBoost model building process. 16](#_Toc182332995)

[**Table H in S1 Text.** The optimized hyperparameters set the main model obtained after random search 17](#_Toc182332996)

[SHAP Dependency plots for climate variables 18](#_Toc182332997)

[**Fig O in S1 Text.** Plots of SHAP values for climate variables. 18](#_Toc182332998)

[SHAP Dependency plots for land use variables 19](#_Toc182332999)

[**Fig P in S1 Text.** Plots of SHAP values for land use variables. 19](#_Toc182333000)

# **Section 1: Sand fly collection**

## Trap types


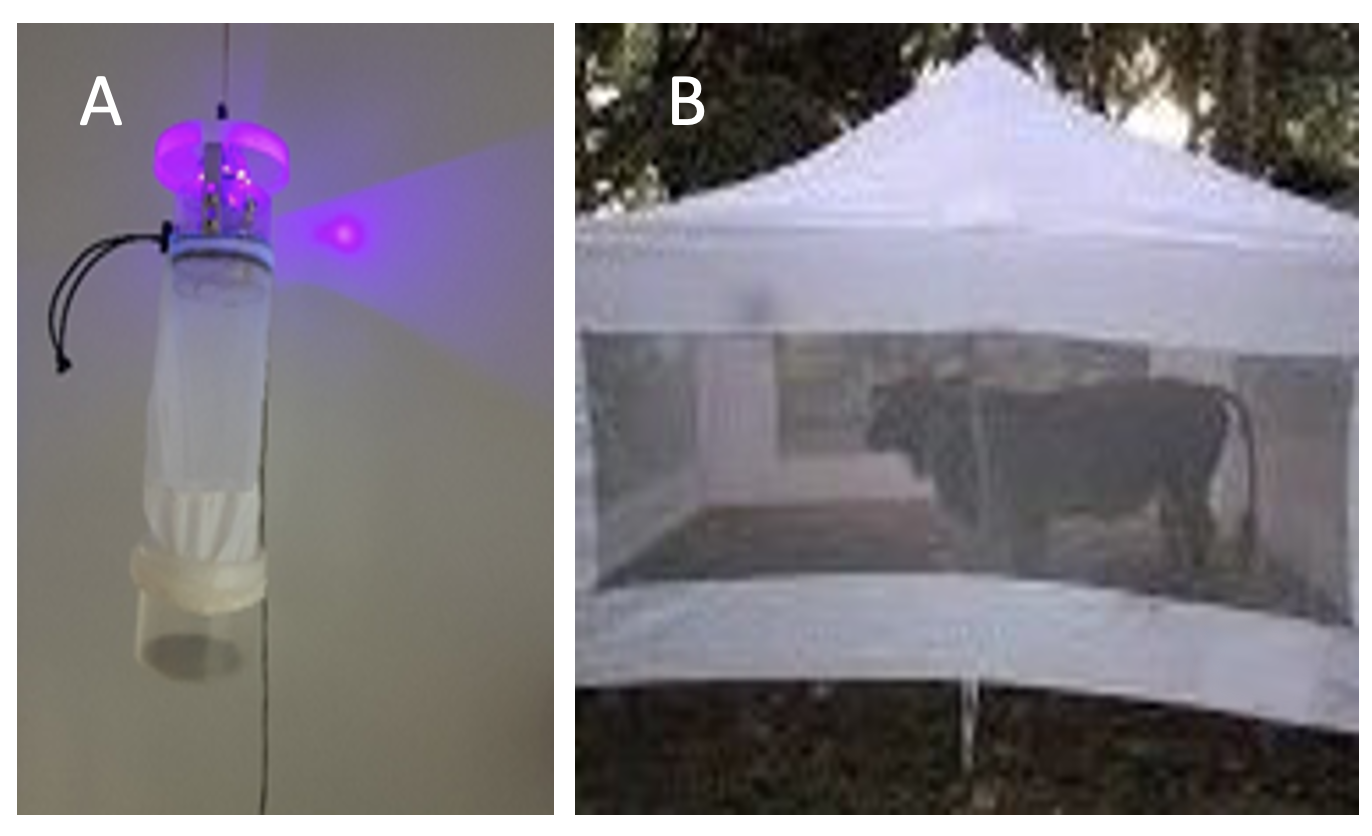


**Fig A in S1 Text.** Sand fly trap types. a) UV LED CDC traps and b) Cattle-baited net traps (CBNT) used to collect adult sand flies from March 2018 to February 2020 at ten sentinel sites over twenty-four months

## The Sampling Locations

The Fig B demonstrates the placement of the light traps and cattle bated traps along with the topological features in a sampling site.


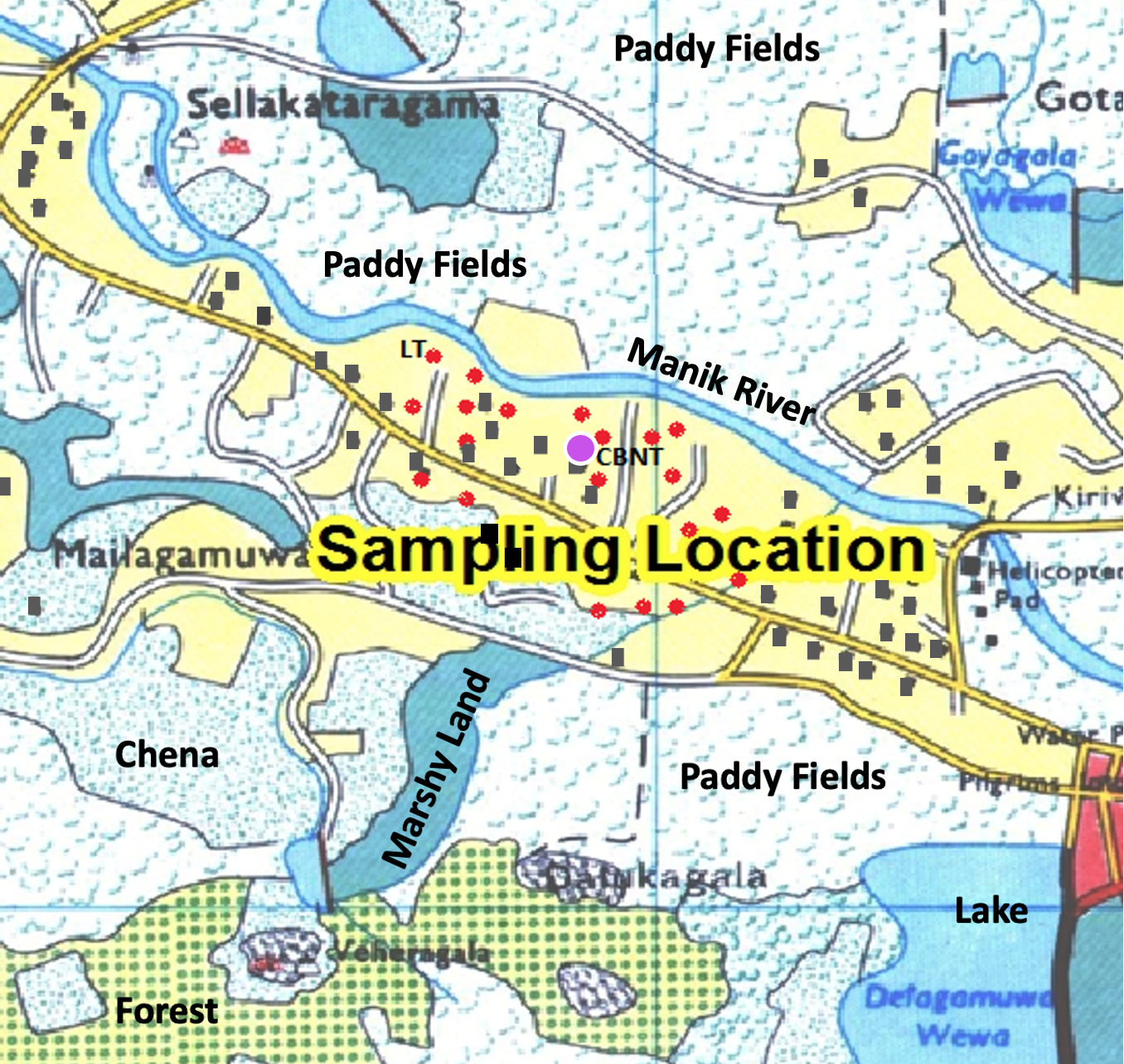


**Fig B in S1 Text.** Placement of the light traps and cattle bated traps. The placement of cattle baited net traps (CBNT - indicated in purple colour dots) and light traps LTs (indicated in red colour dots) in Kataragama sentinel site as an example. The black dots represent the homesteads. The neighbourhood typological features including areas of paddy cultivation, chena, forests, lakes, marshy lands and rivers are highlighted. This schematic diagram was created based on the base map shapefile obtained from the Humanitarian Data Exchange. The data is licensed under Creative Commons Attribution International Governmental Organization (CC BY-IGO). Neighbourhood typological features are added but no modifications to the original map have been made. Link to the base layer of the map <https://data.humdata.org/dataset/sri-lanka-administrative-levels-0-4-boundaries>

# **Section 2: Exploratory data analysis**

**
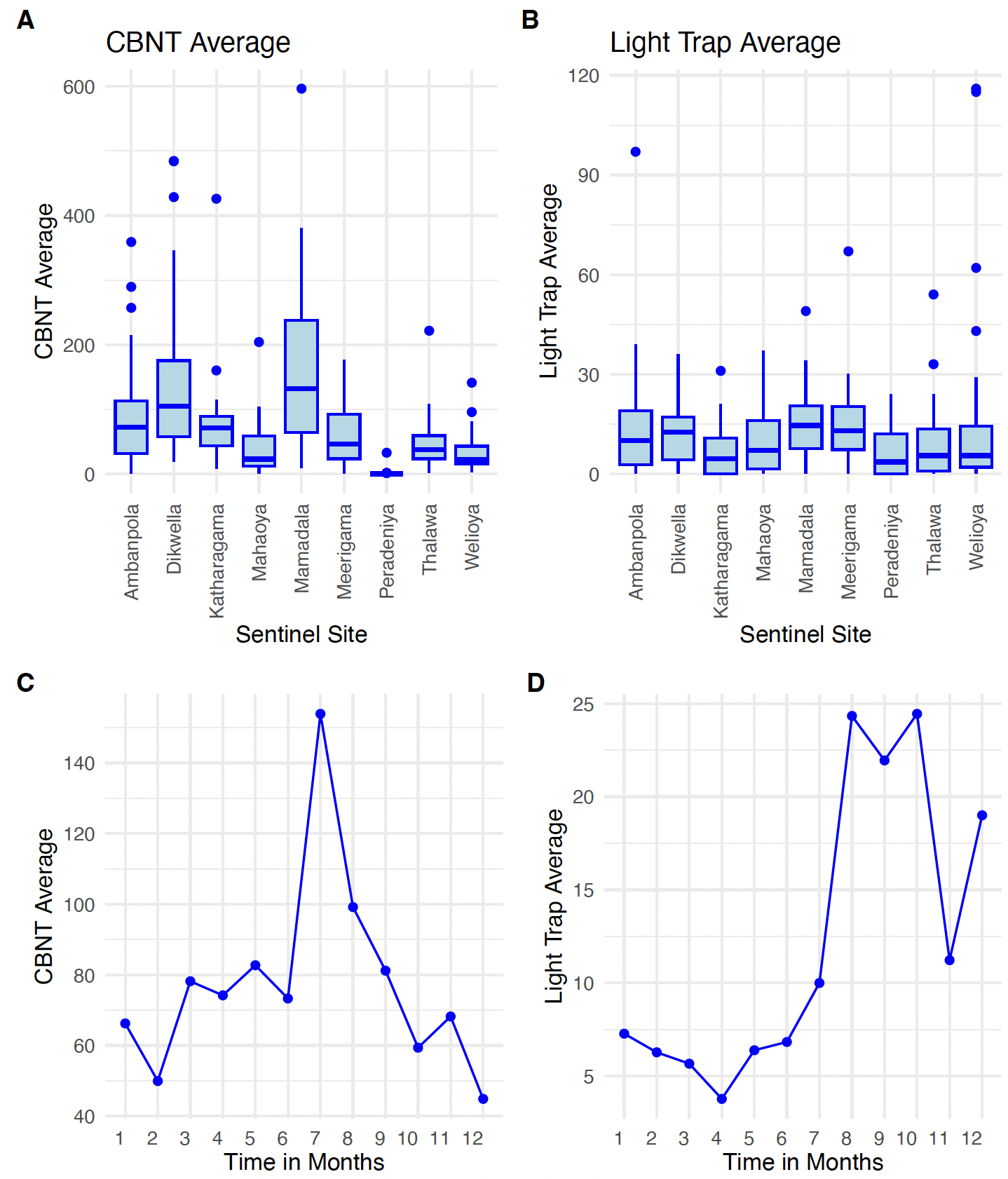
**

**Fig C in S1 Text.** Spatial variability and seasonality of sand fly counts. Panel A and B show the distribution of cattle baited net traps (CBNT) and average counts per light traps (UV LED CDC traps), respectively, across various sentinel sites over 24 months, highlighting spatial variability. Panel C and D present temporal trend averaged across all sentinel sites showing the seasonality. The x-axis in panel A and B represents sentinel sites, C and D represents time in months, while the y-axis displays sand fly count values.

**Table A in S1 Text.** Summary statistics of monthly sand fly density by sentinel site. The table provides summary statistics of sand fly counts, including minimum, maximum, mean, median, and standard deviation, to reflect the variability across individual months for each sentinel site.

| **Summary Statistics for CBNT** | | | | | |
| --- | --- | --- | --- | --- | --- |
| **Sentinel Site** | **Minimum** | **Maximum** | **Mean** | **Median** | **SD** |
| Ambanpola | 0 | 359 | 96.2 | 72.3 | 93.6 |
| Dikwella | 19 | 485 | 160.8 | 104.5 | 145.1 |
| Katharagama | 8 | 426 | 79.7 | 70.8 | 82.5 |
| Mahaoya | 0 | 204 | 38.7 | 22.8 | 45.1 |
| Mamadala | 9 | 597 | 176.5 | 132.0 | 144.0 |
| Meerigama | 0 | 177 | 60.2 | 46.0 | 50.7 |
| Peradeniya | 0 | 33 | 1.5 | 0.0 | 6.6 |
| Thalawa | 2 | 222 | 49.7 | 37.0 | 47.0 |
| Welioya | 3 | 141 | 35.2 | 21.5 | 33.7 |
| **Summary statistics for Light trap** | | | | | |
| **Sentinel Site** | **Minimum** | **Maximum** | **Mean** | **Median** | **SD** |
| Ambanpola | 0 | 97 | 14.8 | 10.0 | 20.4 |
| Dikwella | 0 | 36 | 12.1 | 12.5 | 9.6 |
| Katharagama | 0 | 31 | 7.1 | 4.5 | 8.4 |
| Mahaoya | 0 | 37 | 9.4 | 7.0 | 9.6 |
| Mamadala | 0 | 49 | 16.1 | 14.5 | 11.6 |
| Meerigama | 0 | 67 | 14.6 | 13.0 | 14.1 |
| Peradeniya | 0 | 24 | 6.9 | 3.5 | 8.5 |
| Thalawa | 0 | 54 | 9.8 | 5.5 | 13.1 |
| Welioya | 0 | 116 | 19.5 | 5.5 | 33.0 |

## **Climate variables**


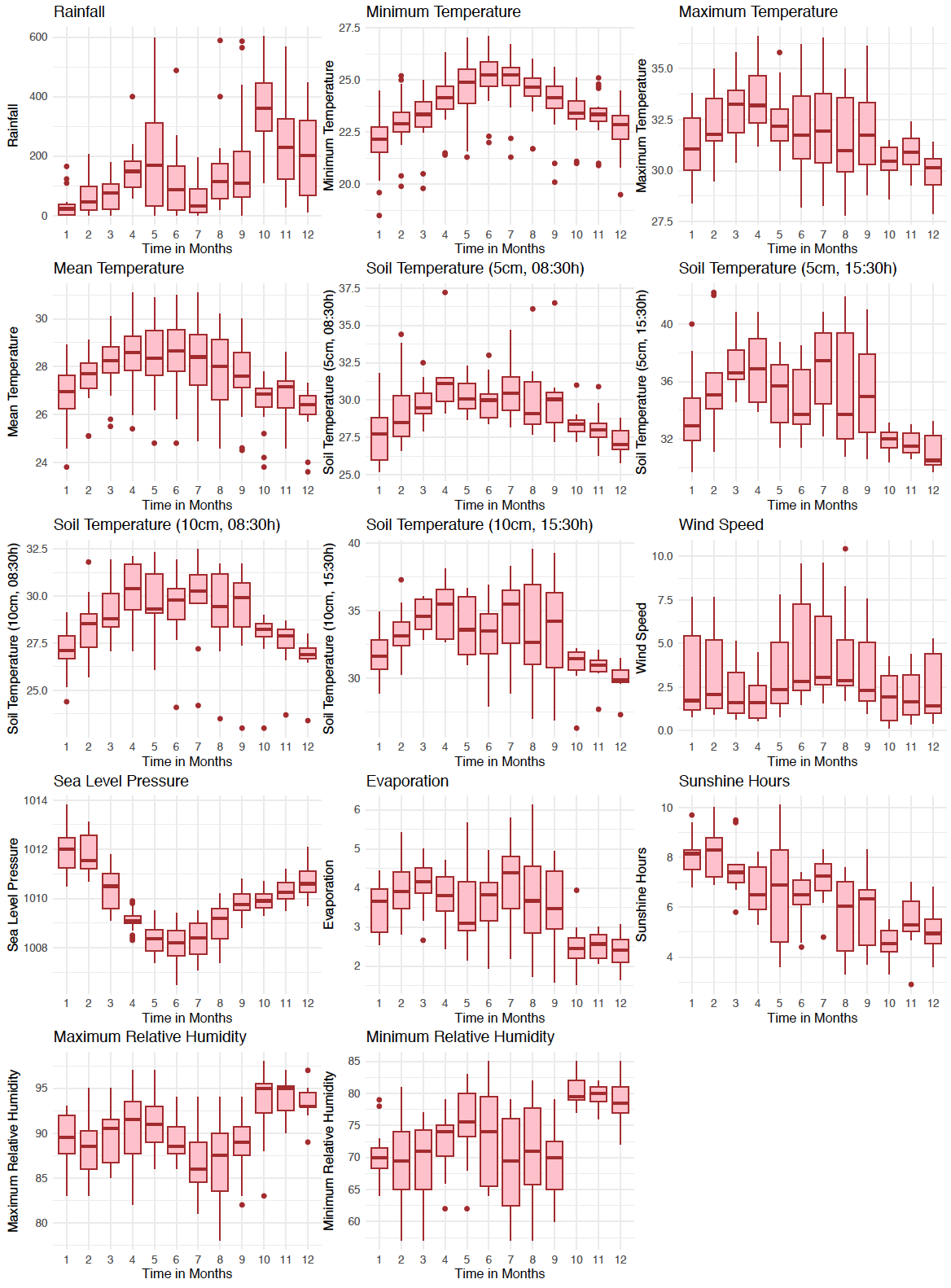


**Fig D in S1 Text.** Box plots showing the seasonality of climate variables averaged across all surveillance settings from March 2018 to Feb 2020. Each boxplot represents the interquartile range of the respective climate variable, with the median value depicted by the line inside the box. The whiskers extend to the minimum and maximum values, excluding outliers represented by individual points. The Y axis denotes the measurement units of each variable, while the X axis indicates the time in months.

## **Correlation between climate variables**


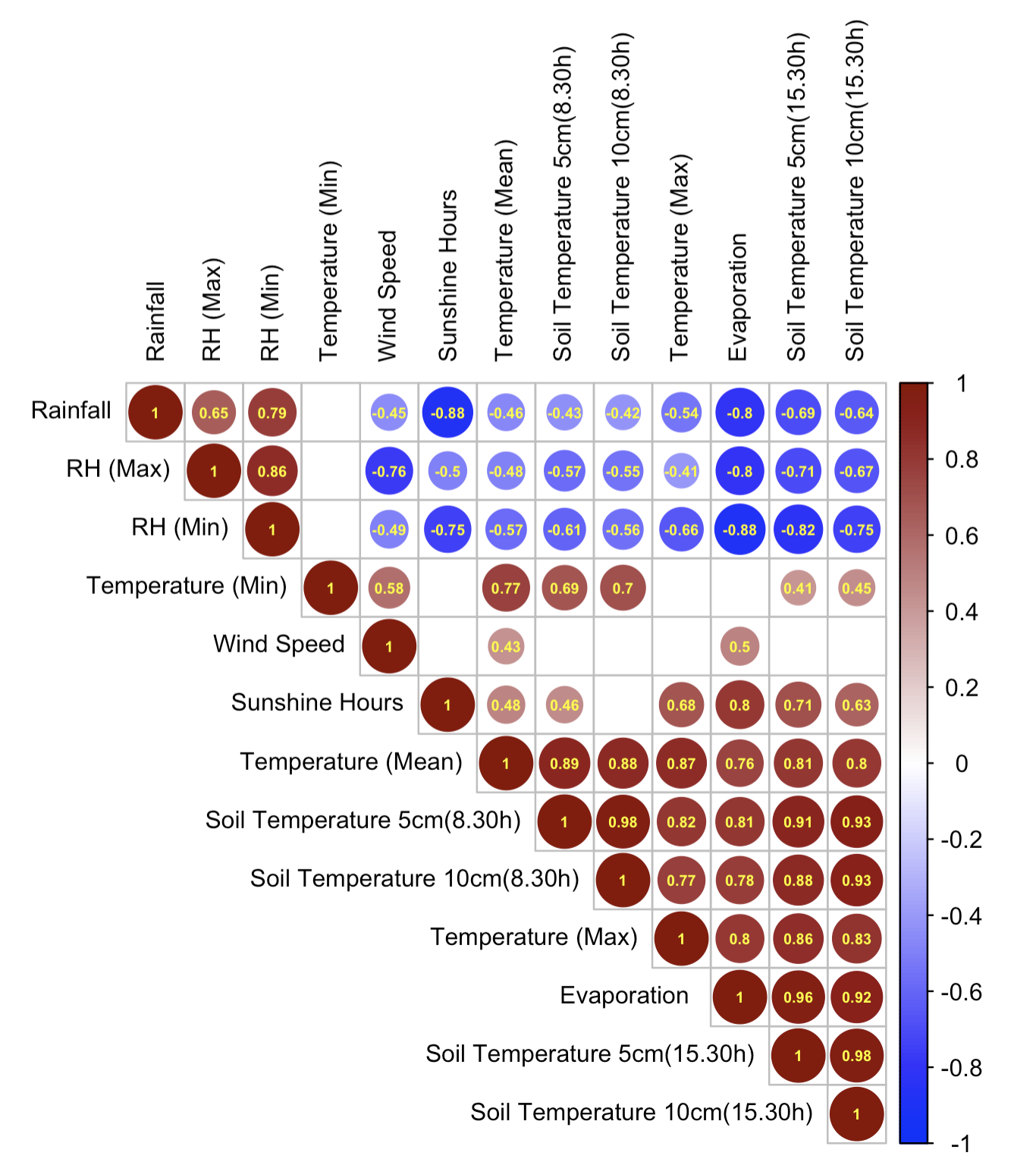


**Fig E in S1 Text.** Correlation between each climatic covariates averaged across all nine monitoring stations from March 2018 to February 2020 in Sri Lanka. Circle size indicates the magnitude of the correlation coefficient, circle colour indicates the direction of the correlation. The scale bar presented in the right margin of the figure indicates the correlation coefficient. Only the statistically significant correlations (p-value <0.05) are shown. RH indicates relative humidity, Min indicates minimum, Max indicates maximum.

## **Non-climate land use variables**

### **Table B in S1 Text.** Distribution of non-climate variables among surveillance sites.

| Variable names | Ambanpola | Dikwella | Mamadala | Katharagama | Mahaoya | Meerigama | Peradeniya | Thalawa | Welioya |
| --- | --- | --- | --- | --- | --- | --- | --- | --- | --- |
| Coconut | 1750.8 | 4823.7 | 277.5 | 0 | 0 | 5749.9 | 0 | 95.9 | 122.9 |
| Chena | 1932.1 | 68.9 | 6352.2 | 4503 | 1271.5 | 0 | 0 | 107.9 | 1414.1 |
| Dense Forest | 69.2 | 1595.9 | 285.6 | 26445.9 | 5589.1 | 346.1 | 690.8 | 399.2 | 38485.6 |
| Homesteads | 25033.8 | 12145.5 | 13309.3 | 3052.2 | 8233.8 | 4138.3 | 12229.7 | 5559.5 | 6189.6 |
| Marshy Land | 99.1 | 0 | 0 | 0 | 19.5 | 0 | 0 | 0 | 773.3 |
| Other Cultivation | 1237.7 | 204.8 | 725.7 | 963.9 | 7988.3 | 319.3 | 0 | 277.1 | 87.8 |
| Paddy | 11515.1 | 2547.8 | 11573.5 | 1606.4 | 1017 | 2335.9 | 2806.7 | 11379.9 | 12964.4 |
| Rocks | 393.8 | 0 | 10.6 | 170.5 | 2094.4 | 34.8 | 44.2 | 1.2 | 21.8 |
| Reservoirs | 1706.7 | 0 | 0 | 2658.8 | 75.8 | 0 | 2.8 | 0 | 0 |
| Scrubs Lands | 5378 | 590.1 | 5142 | 16612.1 | 24598.8 | 293.4 | 898 | 4009.9 | 15754.3 |
| Streams | 94.2 | 12.1 | 294.6 | 467.2 | 522.6 | 31.2 | 232.5 | 2.8 | 603.6 |
| Water body | 3762.6 | 41.7 | 115.9 | 646.1 | 33.3 | 0 | 0 | 0 | 1019 |
| HFI* | 25.22 | 35.32 | 25.72 | 33.1 | 17.85 | 29.04 | 37.61 | 23.34 | 28.71 |

All variables (except HFI) were measured in hectares; *HFI is a dimension-less index.

# **Section 3: Statistical analytical approach**

## **Methods at a glance**

To comprehensively analyze the factors influencing sand fly density, we employed several advanced statistical and machine learning methods. Distributed Lag Non-Linear Models (DLNMs) were used to evaluate the delayed and non-linear effects of weather variables on sand fly populations, allowing us to capture complex time-dependent relationships. A multivariate meta-analysis was conducted to assess the average associations across all study settings, while accounting for heterogeneity in the data. Furthermore, univariable multivariate meta-regression was applied to investigate how different climate zones moderated the relationships between weather and sand fly density.

In addition, machine learning approaches, specifically XGBoost and SHAP (SHapley Additive exPlanations), were integrated to handle the highly correlated and non-linear nature of our exposure variables. SHAP, an explainable AI (exAI) method, was particularly useful for interpreting the model's predictions by quantifying the relative contributions of both climate and non-climate variables at each site. The stepwise analytical procedure is given the Box 1.

### **Box A in S1 Text:** Statistical Analytical Approach

**Box A in S1 Text: Statistical Analytical Approach**

**Methods Used:**

1. Distributed Lag Non-Linear Models (DLNMs)

- Purpose: To assess the delayed (lagged) and non-linear association between weather variables and sand fly density in surveillance sites
- Main statistical package: ‘*dlnm’*

1. Multivariate meta-analysis

- Purpose: To quantify average association across all the study settings while explaining the heterogeneity.
- Main statistical package: ‘*mvmeta’*

1. Univariable multivariate meta regression

- Purpose: To quantify the moderator effect of climate zones on the observed association between weather and sand fly density
- Main statistical package: ‘*mvmeta’*

1. Machine learning: XGBoost and SHAP

- Purpose: To identify the relative contribution and the impact of climate and non-climate variables on sand fly density across the surveillance sites while handling highly correlated non-linear nature of the exposure variables. SHAP, an explainable AI (exAI) method was used to interpret the XGBoost model output.
- Main statistical package: ‘*xgboost’*, ‘*shap’*

All the analytical steps were implemented in R statistical environment

# **Section 4: Distributed Lag Nonlinear Models (DLNM)**

### **Table C in S1 Text.** Sum of the Q-AIC values obtained by the first stage models for LT per trap, CBNT per trap and LT monthly total for each weather variable evaluated.

| Exposure variable | Number of study Settings | LT average | CBNT average | LT monthly total |
| --- | --- | --- | --- | --- |
| Rainfall | 9 | 48.5 | 7763.7 | 1811.5 |
| Soil Temperature (10cm at 8.30 hour) | 9 | 15.8 | 5470.3 | 806.3 |
| Soil Temperature (10cm at 15.30 hour) | 9 | 22.5 | 4927.2 | 873.1 |
| Soil Temperature (5cm at 8.30 hour) | 9 | 15.5 | 5566.1 | 815.8 |
| Soil Temperature (5cm at 15.30 hour) | 9 | 24.3 | 4872.1 | 890.0 |
| Maximum Temperature (ambient) | 9 | 28.3 | 5594.5 | 1141.1 |
| Mean Temperature (ambient) | 9 | 21.1 | 6590.2 | 1110.8 |
| Minimum Temperature (ambient) | 9 | 24.6 | 5614.4 | 959.6 |
| Relative Humidity (Mean) | 9 | 7.2 | 7193.5 | 569.5 |
| Relative Humidity (Maximum) | 9 | 8.4 | 4247.2 | 419.4 |
| Relative Humidity (Minimum) | 9 | 9.3 | 5754.5 | 760.7 |
| Wind Speed | 9 | 12.7 | 3319.1 | 846.4 |
| Sunshine hours* | 5 | 9.9 | 6599.5 | 650.7 |
| Evaporation | 9 | 21.8 | 6840.0 | 961.2 |

*****Sunshine hours were available only for five sites (Mamadala, Thalawa, Ambanpola, Katharagama and Dikewell).

### **Table D in S1 Text**. Definitions of the cross-basis matrix for the selected first stage models.

| **Variable** | **Boundary Knots**  **(lower, upper)** | **Internal Knots for variable** | **Spline (DF) for Variable** | **Spline (DF) for Lag** | **Spline (DF) for Time** |
| --- | --- | --- | --- | --- | --- |
| **Rainfall** (mm/month) | 5.9, 524.9 | 80, 150 | Natural Cubic (3) | Natural Cubic (2) | Natural Cubic (2) |
| **Temperature (^o^C)** | 29.2, 34.5 | 29, 30.5 | Natural Cubic (1) | Natural Cubic (2) | Natural Cubic (2) |
| **Soil Temperature (^o^C)** | 25.9, 31.8 | 28, 30 | Natural Cubic (2) | Natural Cubic (2) | Natural Cubic (2) |
| **Relative Humidity** | 72, 89 | 77, 82 | Natural Cubic (1) | Natural Cubic (2) | Natural Cubic (2) |
| **Sunshine Hours** | 3.5, 9.0 | 5 | Natural Cubic (2) | Natural Cubic (2) | Natural Cubic (2) |
| **Evaporation** | 2, 5.2 | 3, 4 | Natural Cubic (3) | Natural Cubic (2) | Natural Cubic (2) |
| **Wind Speed** | 0.9, | 2 | Natural Cubic (1) | Natural Cubic (2) | Natural Cubic (2) |

The positioning of knots, spline function, and degrees of freedom (DF) utilized in defining cross-basis matrices for climate variables and their lags, as well as the spline and degrees of freedom (DF) employed in the time function of the first-stage surveillance site-specific models, are specified.

## **Full Spectrum of Exposure-Lag-Response Associations**


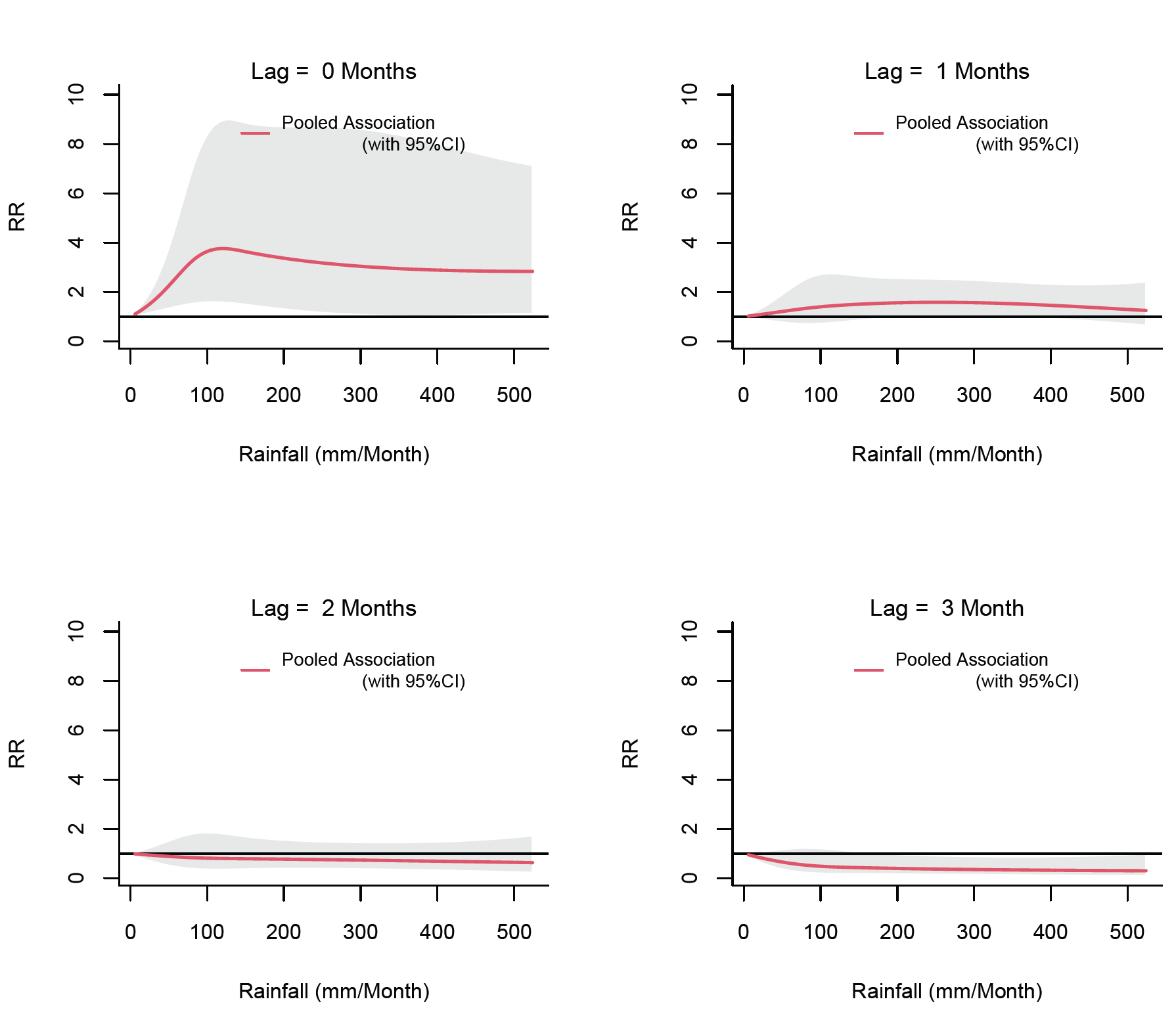


**Fig F in S1 Text.** Relative risk (RR) of leishmaniasis vector activity (measured by LT) by rainfall at a lag of 0 to 3 months. The exposure-response functions at each lag were predicted from the pooled exposure–response function obtained from the meta-analysis for all nine-surveillance site in Sri Lanka, 2018–20. Shaded areas are 95% CIs. Relative risks were calculated with reference to the risk at a rainfall value of 0 mm per month.


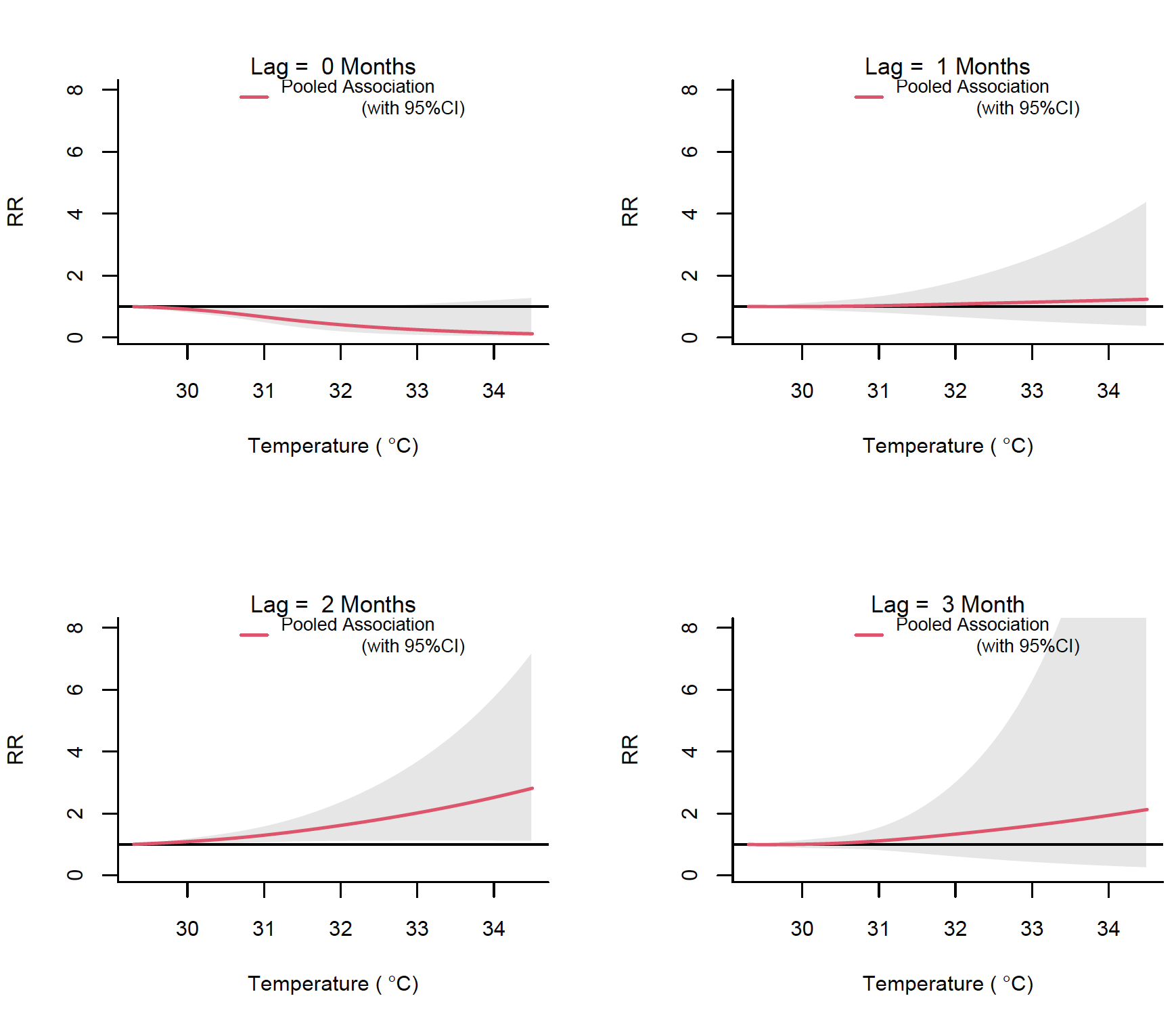


**Fig G in S1 Text.** Relative risk (RR) of leishmaniasis vector activity (measured by LT per trap) by ambient temperature (maximum temperature) at a lag of 0 to 3 months. The exposure-response functions at each lag were predicted from the pooled exposure–response function obtained from the meta-analysis for nine-surveillance sites in Sri Lanka, 2018–20. Shaded areas are 95% CIs. Relative risks were calculated with reference to the risk at a monthly maximum temperature of 29.3^o^C.


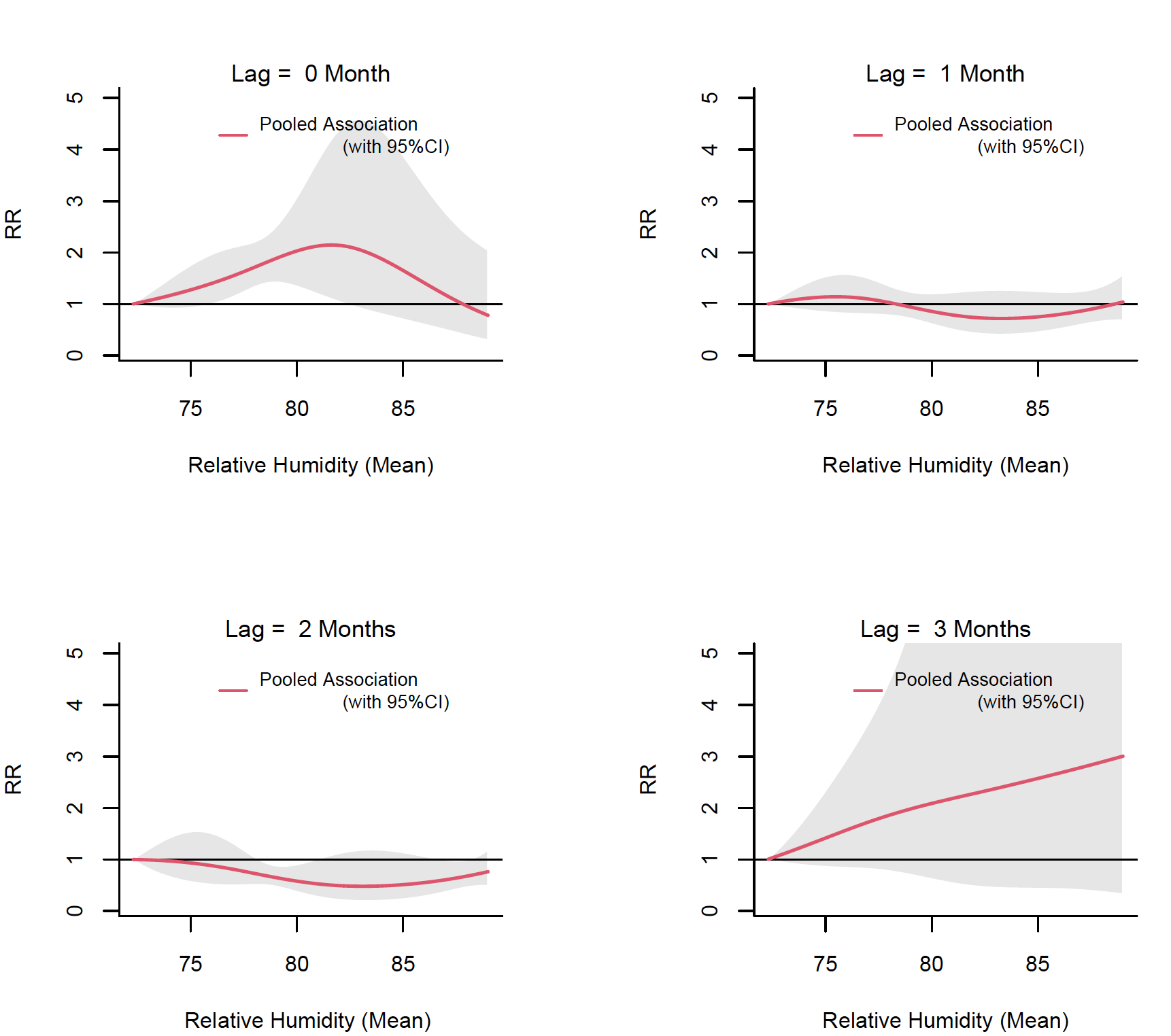


**Fig H in S1 Text.** Relative risk (RR) of leishmaniasis vector activity (measured by LT per trap) by average relative humidity at a lag of 0 to 3 months. The exposure-response functions at each lag were predicted from the pooled exposure–response function obtained from the meta-analysis for all 9 surveillance sites in Sri Lanka, 2018–20. Shaded areas are 95% CIs. Relative risks were calculated with reference to the risk at a monthly average relative humidity of 72.25.


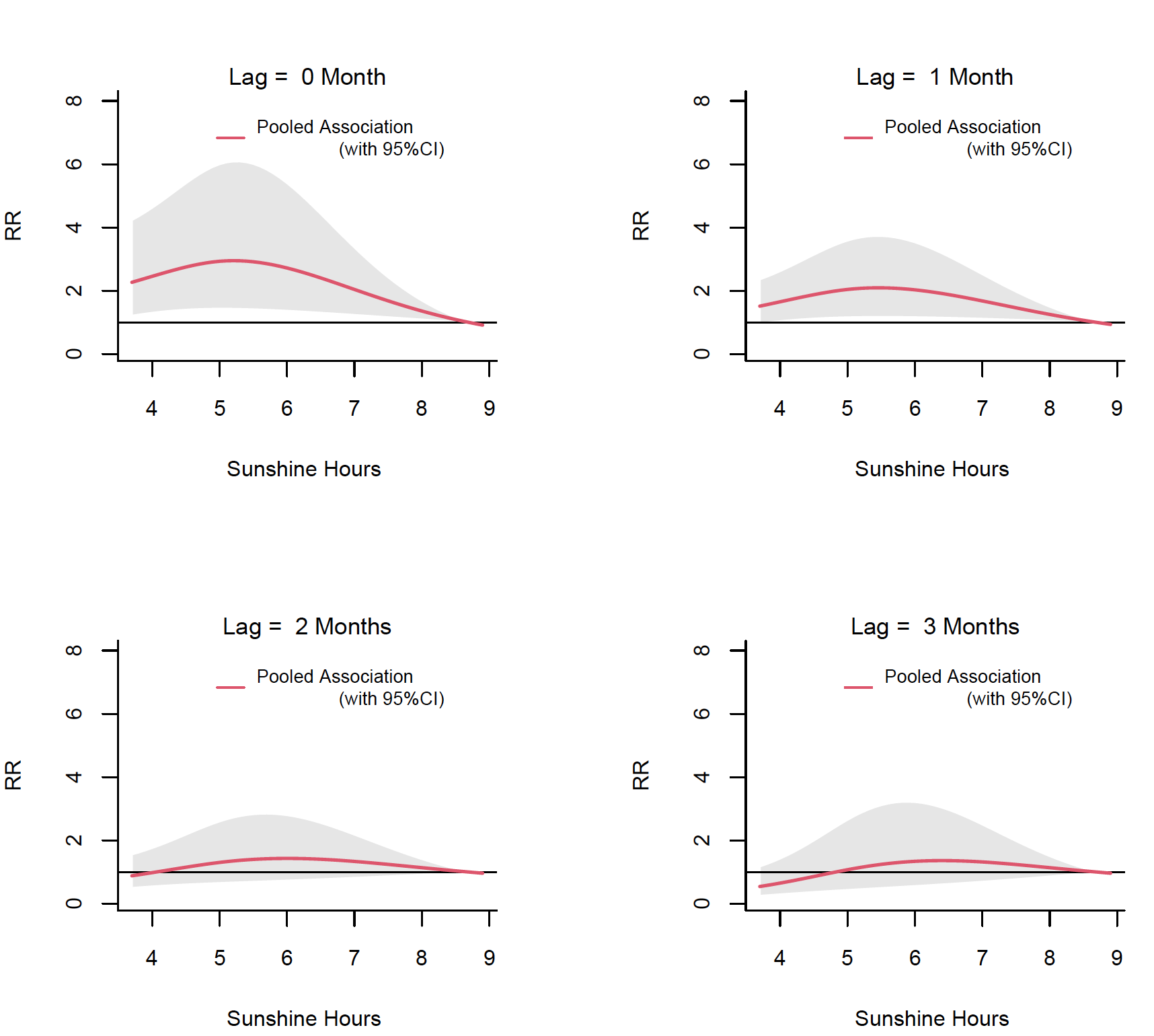


**Fig I in S1 Text.** Relative risk (RR) of sand fly vector density (measured by LT) by sunshine hours at lag of 0 to 3 months. The pooled association (red solid line) was obtained from the meta-analysis of five surveillance sites in Sri Lanka. The grey zone represents the 95% confident interval. Reference was set at the monthly average of daily sunshine hours of 8.7.


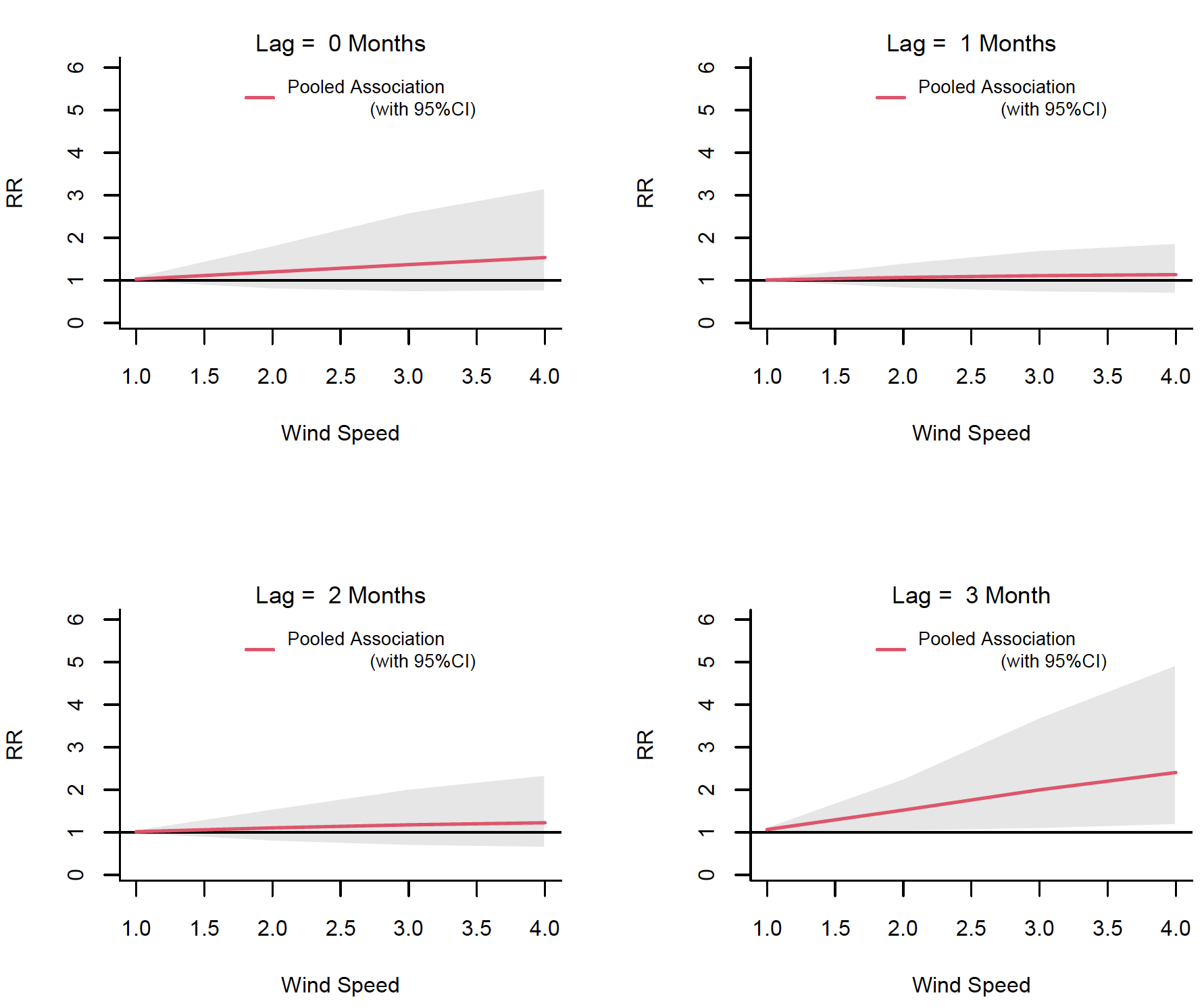


**Fig J in S1 Text.** Relative risk (RR) of sand fly vector density (measured by LT per trap) by wind speed at a lag of 0 to 3 months. The exposure-response functions at each lag were predicted from the pooled exposure–response function obtained from the meta-analysis for seven-surveillance sites in Sri Lanka, 2018–20. Shaded areas are 95% CIs. Relative risks were calculated with reference to the risk at a monthly average wind speed of 1.


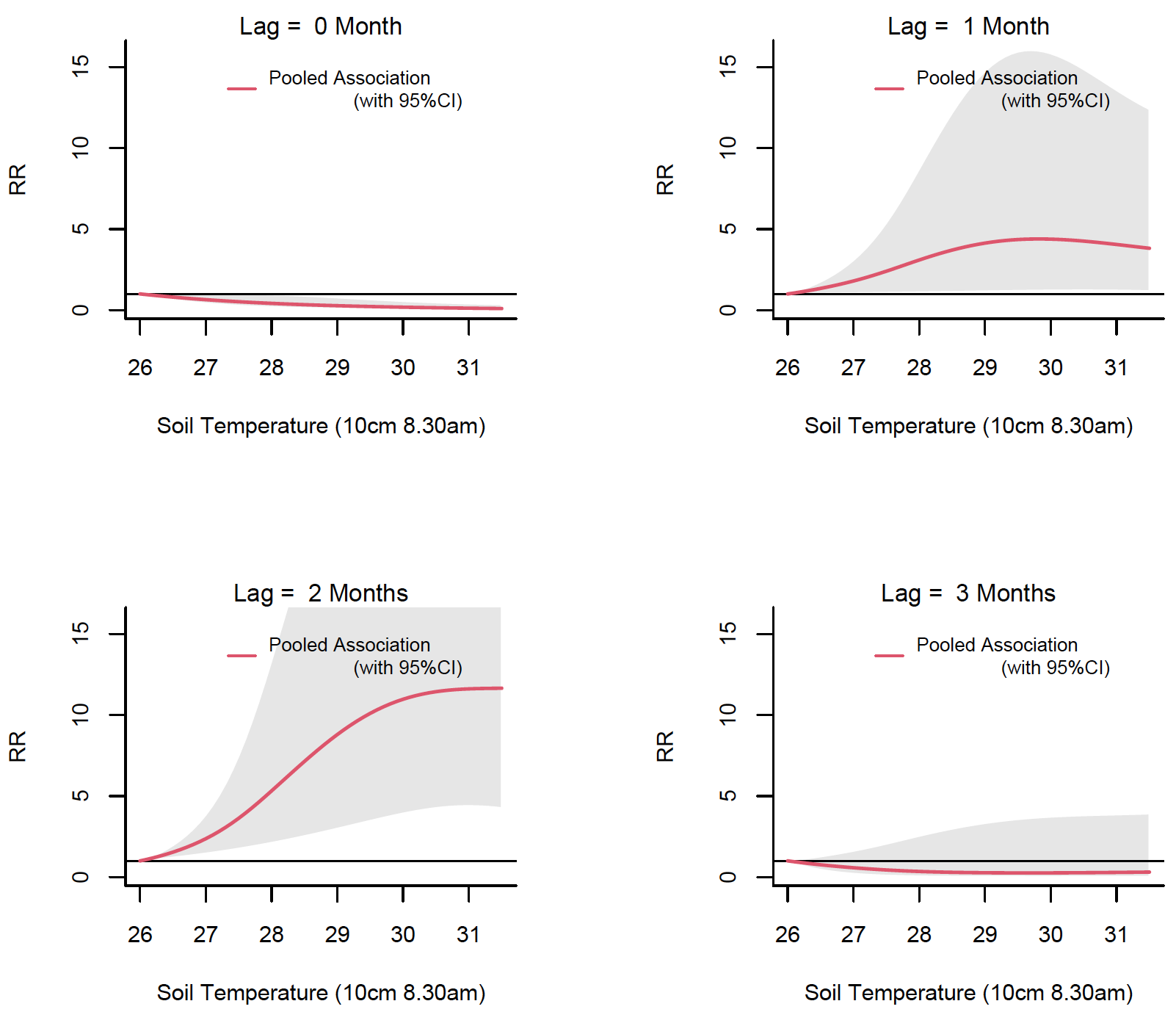


**Fig K in S1 Text.** Relative risk (RR) of sand fly vector density (measured by LT per trap) by soil temperature measured at 10cm form the surface in morning hours (8.30am) at a lag of 0 to 3 months. The exposure-response functions at each lag were predicted from the pooled exposure–response function obtained from the meta-analysis for six-surveillance sites in Sri Lanka, 2018–20. Shaded areas are 95% CIs. Relative risks were calculated with reference to the risk at a soil temperature of 26^0^C.


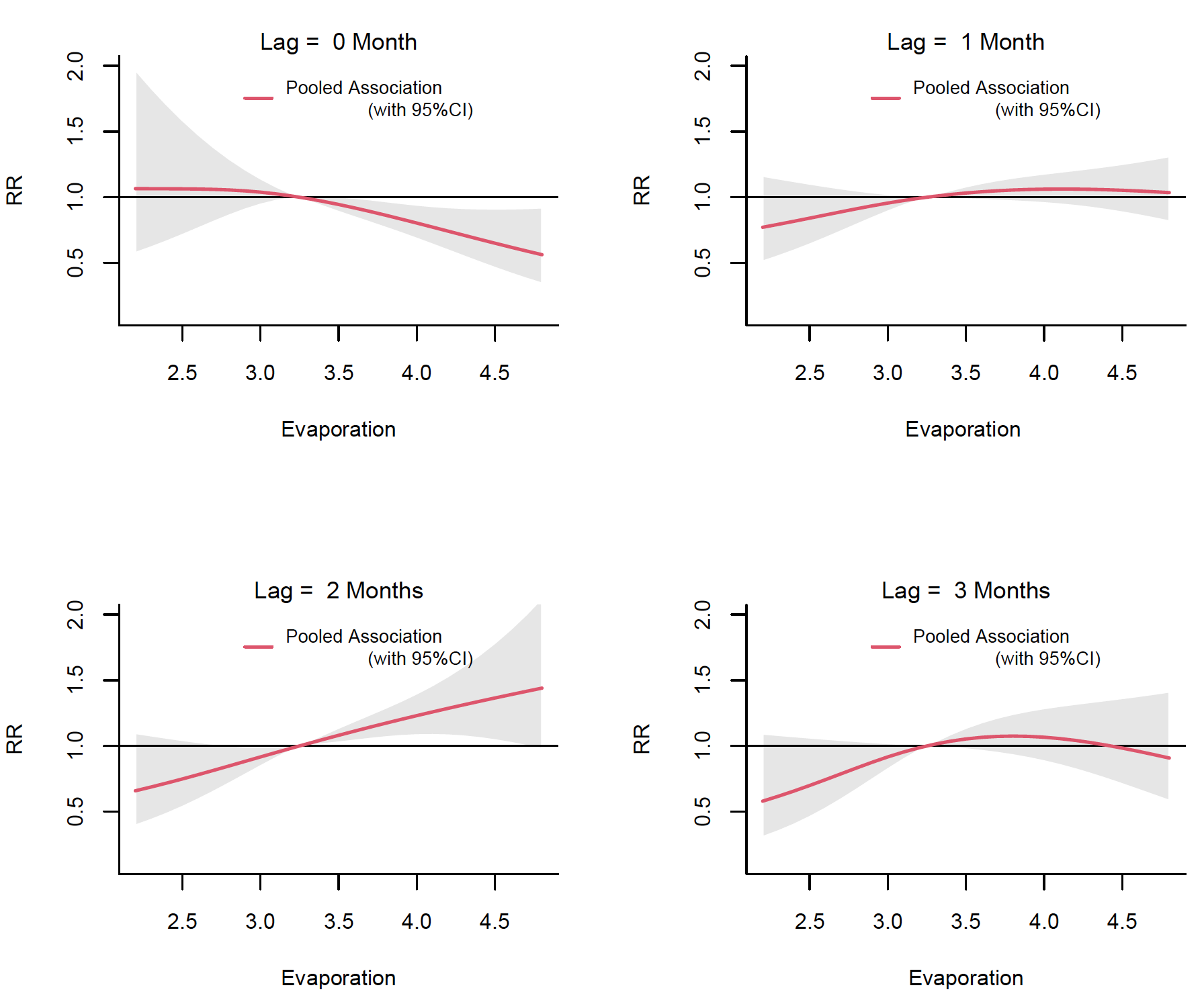


**Fig L in S1 Text.** Relative risk (RR) of sand fly vector density (measured by LT per trap) by evaporation values at a lag of 0 to 3 months. The exposure-response functions at each lag were predicted from the pooled exposure–response function obtained from the meta-analysis for six-surveillance sites in Sri Lanka, 2018–20. Shaded areas are 95% CIs. Relative risks were calculated with reference to the risk at a monthly average evaporation of 3.3.

**Table E in S1 Text.** Quantification of divisional heterogeneity of the association between weather variables and the LT per trap index obtained by the second stage multi-variate meta-analysis. Q test of heterogeneity, the corresponding degree of freedom, p-values and the *I^2^* statistics for each weather variable are shown.

| **Weather variables and their respective lag period (months)** | **Q test** | **df** | **p-value** | ***I^2^* statistic** |
| --- | --- | --- | --- | --- |
| Rainfall lag 0 | 47.1 | 24 | 0.003 | 49.0% |
| Soil Temperature (10cm at 8.30 hour) lag 0 | 11.8 | 10 | 0.292 | 15.9% |
| Soil Temperature (10cm at 8.30 hour) lag 2 | 18.9 | 10 | 0.040 | 47.3% |
| Maximum Temperature (ambient) lag 0 | 21.7 | 16 | 0.153 | 26.3% |
| Maximum Temperature (ambient) lag 2 | 27.3 | 16 | 0.038 | 41.4% |
| Mean Temperature (ambient) lag 0 | 19.7 | 16 | 0.145 | 28.9% |
| Mean Temperature (ambient) lag 2 | 19.7 | 16 | 0.138 | 29.1% |
| Minimum Temperature (ambient) lag 0 | 8.4 | 16 | 0.752 | 1.0% |
| Minimum Temperature (ambient) lag 2 | 6.6 | 16 | 0.879 | 1.0% |
| Relative Humidity (Mean) lag 0 | 29.7 | 15 | 0.012 | 49.6% |
| Relative Humidity (Mean) lag 2 | 37.3 | 15 | 0.001 | 59.8% |
| Wind Speed lag 0 | 12.1 | 12 | 0.431 | 1.5% |
| Wind Speed lag 3 | 14.0 | 12 | 0.297 | 14.6% |
| Sunshine hours lag 0 | 2.7 | 8 | 0.950 | 1.0% |
| Sunshine hours lag 1 | 5.9 | 8 | 0.649 | 1.0% |
| Evaporation lag 0 | 11.0 | 10 | 0.339 | 11.0% |
| Evaporation lag 2 | 16.6 | 10 | 0.081 | 40.0% |

## **Exposure-lag-response association between weather variables and sand fly density measured by cattle baited net traps (CBNT)**


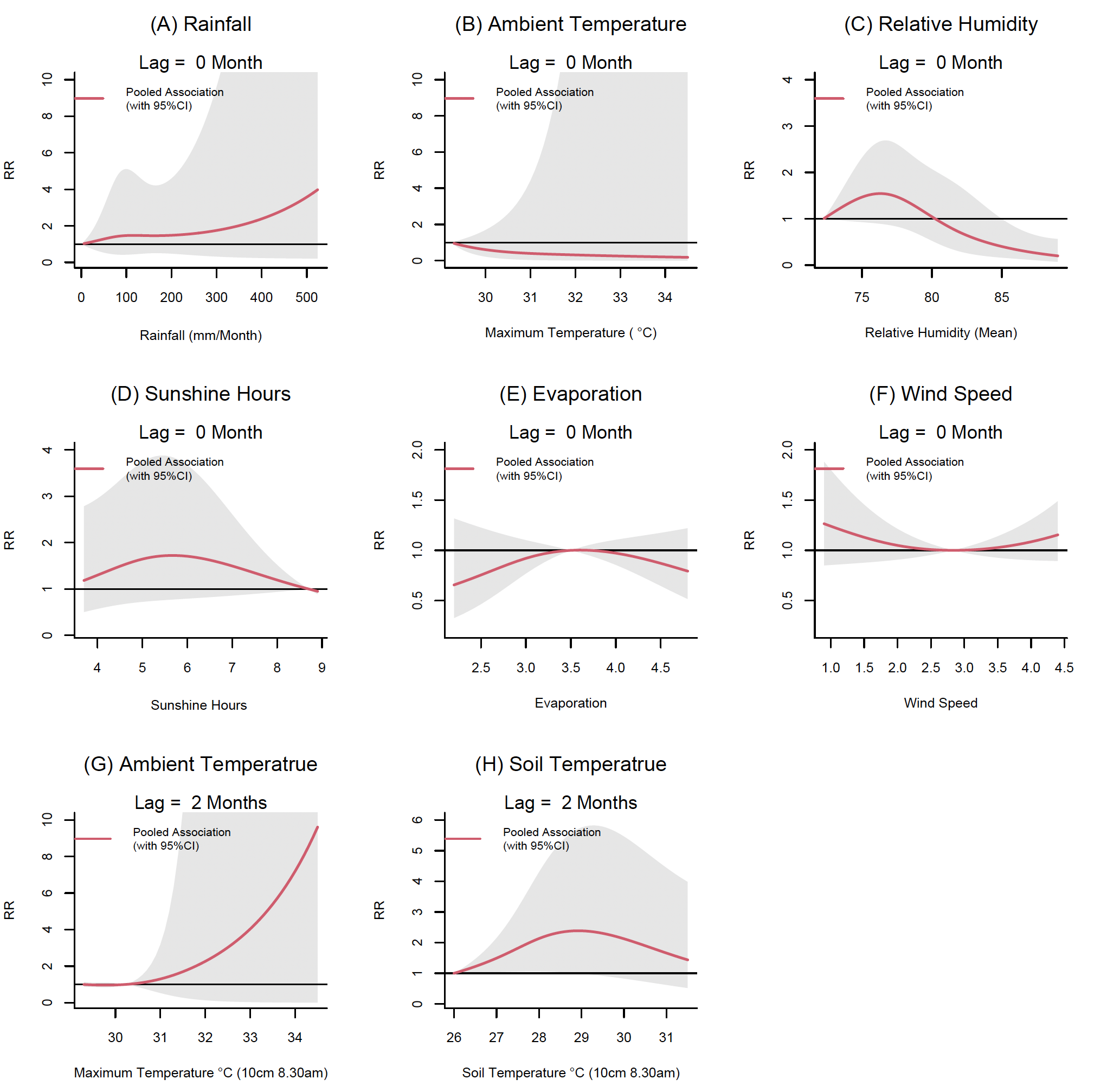


**Fig M in S1 Text.** Weather and sand fly density measured by CBNT. Relative risk (RR) of leishmaniasis vector density (measured by CBNT) by rainfall (A), ambient temperature (maximum temperature) (B), average relative humidity (C), sunshine hours (D), evaporation (E), wind speed (F) at a lag of 0 months, ambient temperature at lag of 2 moths (G) and soil temperature at lag of 2 months (H). The exposure-response functions at lag of 0 month were predicted from the pooled exposure-response function obtained from the meta-analysis for all surveillance sites in Sri Lanka, 2018–20. Shaded areas are 95% CIs. Relative risks were calculated with reference to the risk at a rainfall value of 0 mm per month, maximum temperature of 29.30C, average relative humidity of 72.25, average evaporation of 3.3mm and wind speed of kmh^-1^.

# **Section 5: Moderator effect of climate zones on weather-Sand Fly association**

The weather variables having statistically significant heterogeneity across study settings were selected for this analysis. Univariable multivariate meta-regression was conducted at the second stage of the multivariate meta-analysis to quantify the moderator effect of climate zones as defined by the annual average rainfall values. Annual average rainfall for each study setting was estimated by the corresponding monthly average rainfalls. The moderator effect of annual average rainfall was estimated for the full range of rainfall values covering the three main climate zones. Wald test statistics was used to test the statistical significance of the moderator effect at a p value of 0.05 and a 95% confidence limit for all selected weather variables. The Table 5 provide the Wald test statistics and compare the Q-test of heterogeneity between the models adjusted for climate zones and those that were not adjusted. The Fig 12 demonstrates the moderator effect of annual average rainfall divided into the corresponding climate zones. The figure provides a comprehensive overview of the direction of moderation for each climate zone on the sand fly association for the full range of weather exposure. All the possible values between the minimum and maximum annual average rainfall values reported across all study settings has been assigned to the corresponding climate zone.

**Table F in S1 Text:** Wald test statistics for the moderator effect of climate zones on weather-sand fly association. Cochran Q-test of heterogeneity and related p value and Wald test statistics along with the corresponding p value obtained for selected weather variables are shown and compared with the unadjusted models.

| **Weather variable** | **Climate adjustment** | **Q test of heterogeneity** | **p-value** | **Wald Test** | **p-value** |
| --- | --- | --- | --- | --- | --- |
| Rainfall | Unadjusted | 47.1 | 0.003 |  |  |
|  | Adjusted | 42.05 | 0.004 | 3.67 | 0.300 |
| Soil temperature lag 2 | Unadjusted | 18.9 | 0.040 |  |  |
|  | Adjusted | 16.83 | 0.032 | 1.57 | 0.456 |
| Ambient Temperature lag 2 | Unadjusted | 27.3 | 0.038 |  |  |
|  | Adjusted | 23.67 | 0.050 | 3.65 | 0.162 |
| Relative humidity lag 0 | Unadjusted | 29.7 | 0.012 |  |  |
|  | Adjusted | 25.44 | 0.013 | 3.64 | 0.303 |


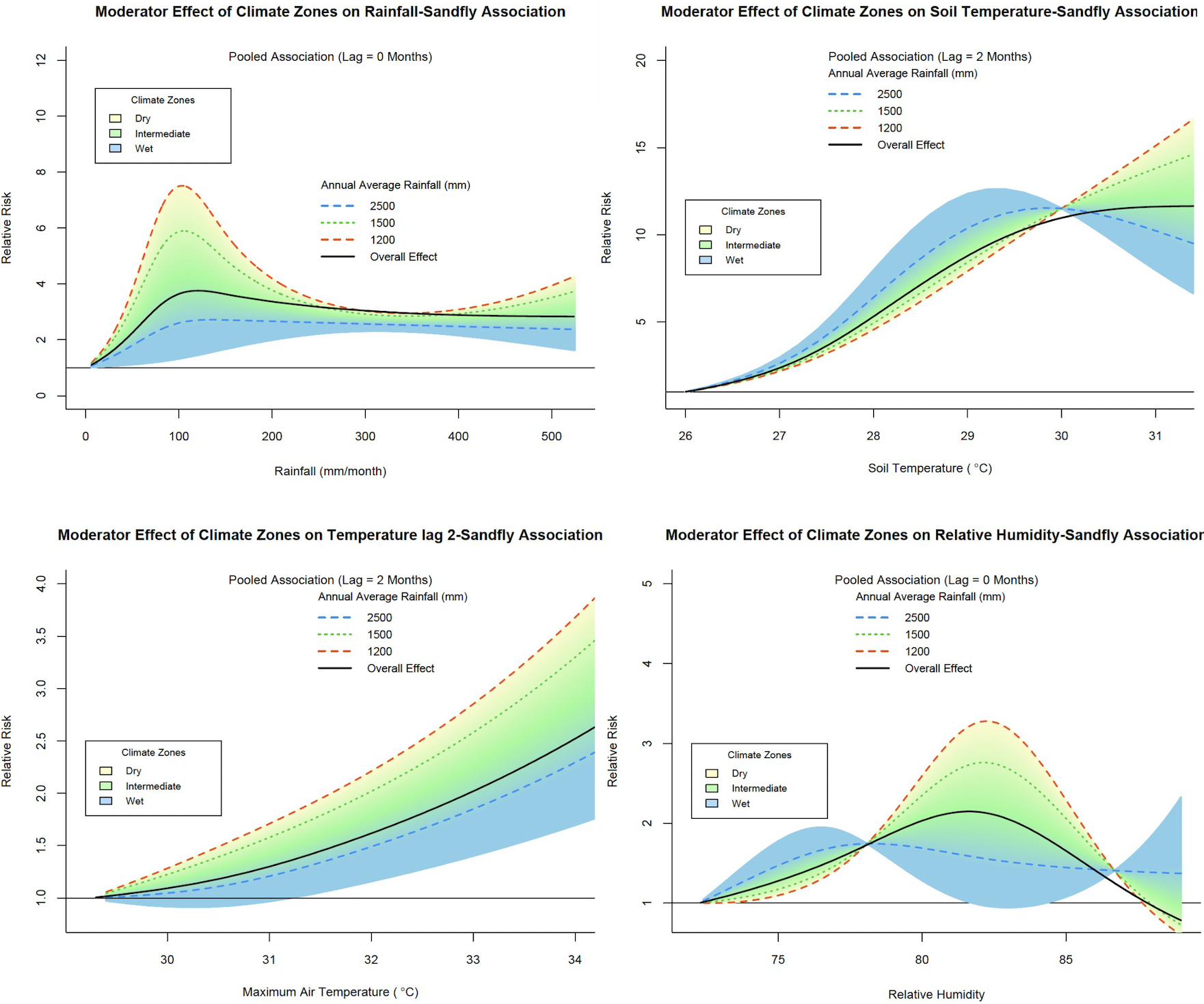


**Fig N in S1 Text.** Moderator effect of climate on weather and Sand Fly density. Each plot demonstrates the relative risk estimated for the full range of annual average rainfall values for each weather variable. The solid black line represents the overall lag specific effect estimated (pool association across all the study settings) during the second stage meta-analysis. The blue, green and red dotted lines represent annual average rainfall values of 2500mm, 1500mm and 1200 mm corresponding to the wet, intermediate and dry zone respectively. The blue-green-orange color band represent the full spectrum of annual average rainfall values distributed across wet, intermediate and dry zones in the study settings.

# **Section 6: Machine learning and XAI**

## **XGBoost model building and validation**

All lagged climate variables, identified using the DLNM approach described above, were incorporated into the XGBoost model, along with the non-climate variables listed in S1 Table 1. The stepwise summary of model implementation is given in the S1 Table 6 below.

### **Table G in S1 Text.** Stepwise summary of XGBoost model building process.

| **Step** | **Description** |
| --- | --- |
| **Model Training** | The XGBoost gradient-boosted tree regression algorithm was used, incorporating 23 climate and non-climate variables. |
| **Hyperparameter Tuning** | A random search algorithm was used to tune hyperparameters: max_depth (tree depth), eta (step size shrinkage), subsample (subsample ratio of training instances), colsample_bytree (subsample ratio of columns for each tree), and min_child_weight (minimum number of instances per tree node). |
| **Cross-Validation** | 5-fold cross-validation was employed to prevent model overfitting. R-squared values were used to assess model performance. |
| **Model Validation** | The model was further validated using Adj-R squared and RMSE metrics. Data was split into training (80%) and test (20%) sets, and this process was repeated 10 times to minimize variability and ensure robust performance estimates. |
| **Feature Ranking with SHAP** | SHAP values were computed for each variable to rank features based on their contribution to the model. The positive and negative impacts of variables on sand fly density were evaluated and visualized in a global feature importance bar diagram and local explanation summary plots. |

First, we trained the model using XGboost gradient-boosted tree regression algorithm using all 23 climate and non-climate variables. To maximize the model’s performance, we used a random search algorithm to tune hyperparameters. Specifically, we tuned max_depth, which defines the maximum depth of a tree, eta, step size shrinkage parameter to prevent overfitting, subsample, a subsample ratio of the training instances, colsample_bytree, a subsample ratio of columns for each tree, and min_child_weight, a minimum number of instances needed to be in each tree node. Details regarding the hyperparameter settings and final optimal parameters can be found in S1 Table 7. We also used the 5-fold cross-validation to ensure the model is not an overfit to the data. The model’s performance was assessed using R-squared values. The model fit was further validated using Adj-R squared and RMSE metrics through a secondary analysis involving random partitioning of the data into training (80%) and test (20%) sets. This cross-validation process was repeated up to ten times to reduce variability between different splits and ensure robust and reliable performance estimates. We then applied SHAP on the best-fit model to rank the features in the order of their contribution. SHAP values for each variable were computed to evaluate their positive and negative impacts on sand fly vector density and presented in a global feature importance bar diagram and local explanation summary plots.

### **Table H in S1 Text.** The optimized hyperparameters set the main model obtained after random search

| **Parameter** | **Optimal Value** |
| --- | --- |
| Number of decision trees (nrounds) | 324 |
| Maximum depth of a tree (max_dapth) | 6 |
| Subsample ratio of columns when constructing each tree  (colsample_bytree) | 0.9899826 |
| Learning rate (eta) | 0.27 |
| Minimum loss reduction (gamma) | 1 |
| Subsample ratio of the training instances (subsample) | 0.7317348 |
| Minimum sum of instance weight (min_child_weight) | 3 |
| L2 regularization term on weights | 0.87 |
| **Validation** | **Value** |
| Training data Adj R^2^ | 0.85 |
| Training data RMSE | 0.38 |
| Test data Adj R^2^ | 0.71 |
| Test data RMSE | 0.64 |

## **SHAP Dependency plots for climate variables**

**
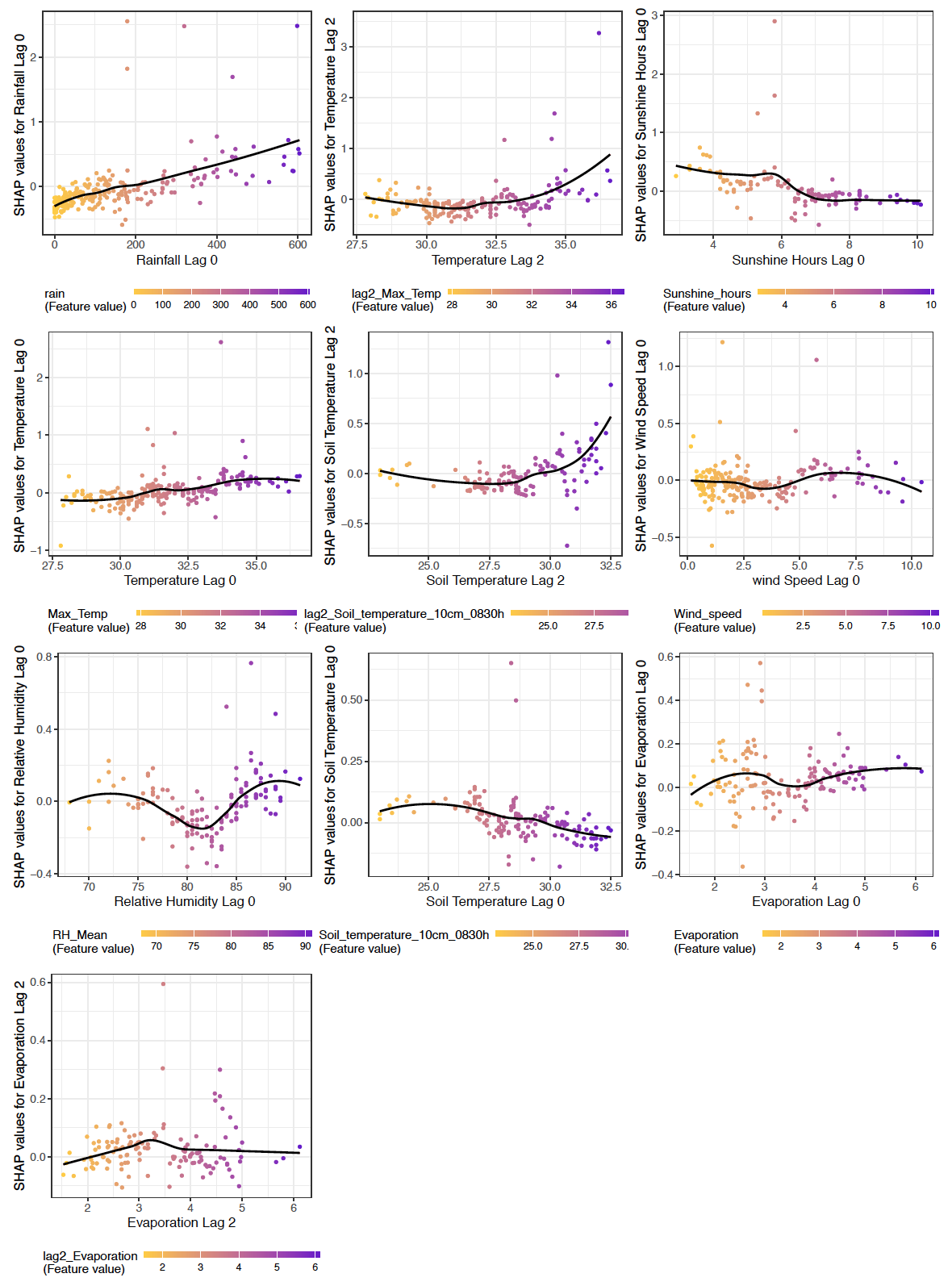
**

**Fig O in S1 Text.** Plots of SHAP values for climate variables. Each plot demonstrates the relationships between the climate variables and sand fly densities as predicted by the XGBoost model. Each dot represents to the SHAP value for the corresponding value of the climate variable. The black color line shows locally estimated scatterplot smoothing.

## **SHAP Dependency plots for land use variables**

**
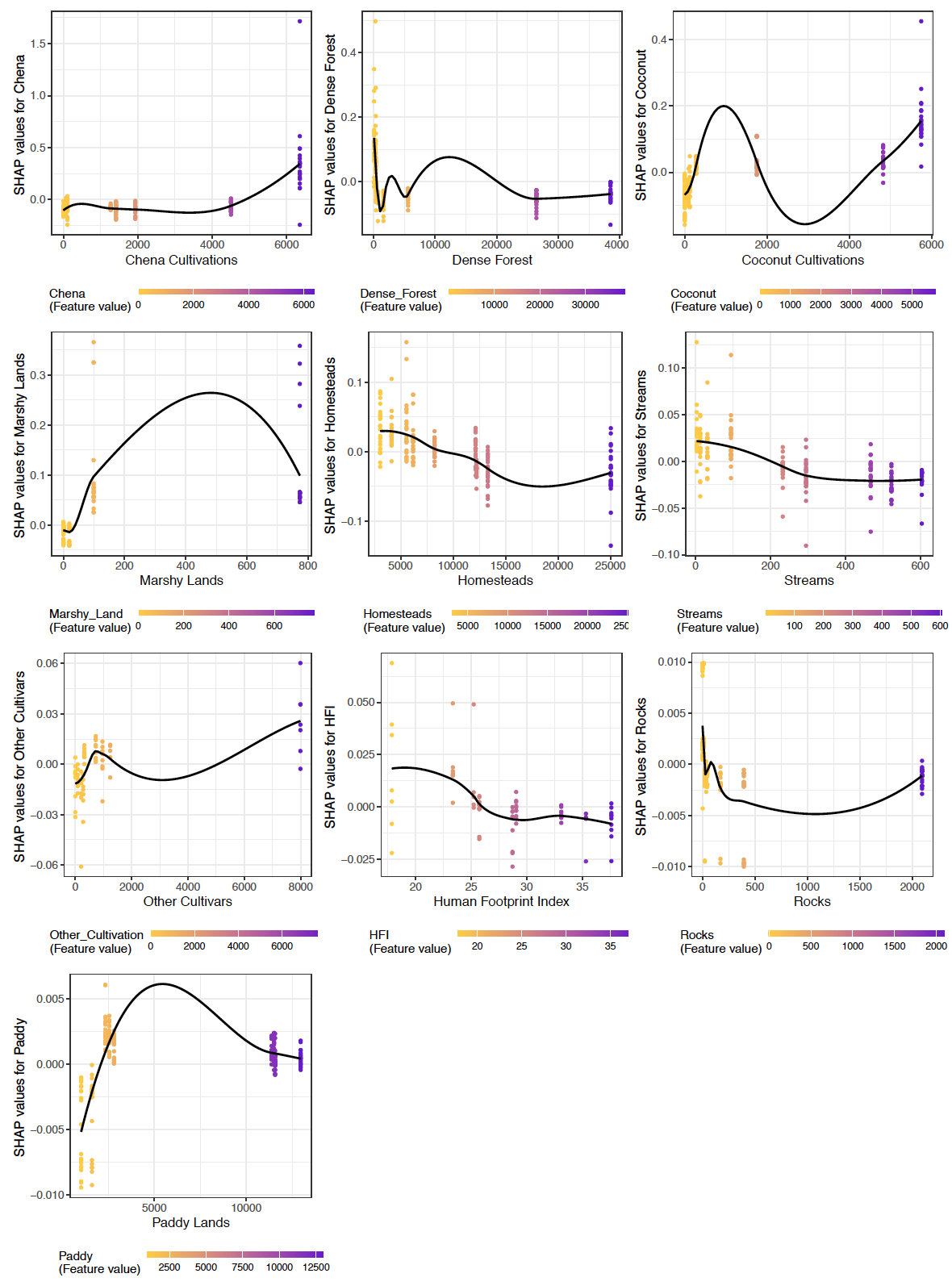
**

**Fig P in S1 Text.** Plots of SHAP values for land use variables. Each plot demonstrates the relationships between the non-climate variables and sand fly densities as predicted by the XGBoost model. Each dot represents to the SHAP value for the corresponding value of the climate variable. The black color line shows locally estimated scatterplot smoothing.
