# Supplementary material for: Impact of climate and land use on the temporal variability of sand fly density in Sri Lanka: A 2-year longitudinal study
Source: PLoS Negl Trop Dis. 2024 Nov 21;18(11):e0012675. doi: 10.1371/journal.pntd.0012675 (PMC11620634; doi:10.1371/journal.pntd.0012675)
Supplement: Attachment — Submitted filename: PNTD-D-24-00582.pdf [file pntd.0012675.s003.pdf]

PLOS Neglected Tropical Diseases

Impact of climatic factors on the temporal variability of sand fly abundance in Sri Lanka: A 2-year longitudinal study

--Manuscript Draft--

**Manuscript Number:**	PNTD-D-24-00582
**Full Title:**	Impact of climatic factors on the temporal variability of sand fly abundance in Sri Lanka: A 2-year longitudinal study
**Short Title:**	Climate variability and sand fly abundance in Sri Lanka
**Article Type:**	Research Article
**Keywords:**	P. argentipes, sand flies, vector, climate, Leishmania, parasite
**Abstract:**	Background<br>Phlebotomine sandflies are the vectors of leishmaniasis. The sand fly abundance tends to be influenced by context-specific climatic and non-climatic factors. Thus, we aimed to understand how these factors drive sand fly density in ten sentinel sites across Sri Lanka.<br>Methodology/Principal Findings<br>We analysed monthly collections of sand flies and climate data from ten sentinel sites representing all geo-climatic zones across Sri Lanka, over 24 months. Site-specific non-climate data was also recorded. The influence of climate and non-climate drivers on sand fly abundance in each site was calculated using distributed lag non-linear models and machine learning, We found that climate plays a major role on sandfly abundance compared to non-climate factors. Increase in rainfall and relative humidity at real time, and ambient temperature and soil temperature with a 2-month lag were associated with a statistically significant increase in sand fly density. The maximum relative risk (RR) was 3.76 (95% CI: 1.58-8.96) for rainfall at 120 mm/month, 2.14 (95% CI: 1.04-4.38) for relative humidity at 82%, 2.81 (95% CI: 1.09-7.35) both at real time. For ambient temperature at 34.5°C, and 11.6 (95%CI; 4.38-30.76) for soil temperature at 31.5oC; latter 2 variables with a 2-month lag period. A similar delayed association was also seen with the rise of soil temperature and evaporation rates. The real-time increase in ambient temperature, sunshine hours, and evaporation rate, however, reduced sand fly burden homogeneously in all study settings. The high density of chena and coconut cultivation, together with low density of dense forests, homesteads, and low human footprint values, positively influenced sandfly densities.<br><br>Conclusions/Significance<br>The findings would enhance understanding of the dynamic influence of environment on sand flies and leishmaniasis spread, laying a foundation for for forecasting of sand fly burden and targeted site-specific interventions for mitigating the growing burden of leishmaniasis, particularly in an era of climate change.
**Additional Information:**	
**Question**	**Response**
**Government Employee**<br><br>Are you or any of the contributing authors an employee of the United States government?<br><br>Manuscripts authored by one or more US Government employees are not copyrighted, but are licensed under a CC0 Public Domain Dedication, which allows unlimited distribution and reuse of the	No - No authors are employees of the U.S. government.

article for any lawful purpose. This is a legal requirement for US Government employees.<br><br>This will be typeset if the manuscript is accepted for publication.	
**Financial Disclosure**<br><br>Enter a financial disclosure statement that describes the sources of funding for the work included in this submission. Review the submission guidelines for detailed requirements. View published research articles from *PLOS Neglected Tropical Diseases* for specific examples.<br><br>This statement is required for submission and **will appear in the published article** if the submission is accepted. Please make sure it is accurate.<br><br>**Funded studies**<br>Enter a statement with the following details:<br>• Initials of the authors who received each award<br>• Grant numbers awarded to each author<br>• The full name of each funder<br>• URL of each funder website<br>• Did the sponsors or funders play any role in the study design, data collection and analysis, decision to publish, or preparation of the manuscript?<br><br>Did you receive funding for this work?	Yes
Please add funding details.<br>as follow-up to "**Financial Disclosure**<br><br>Enter a financial disclosure statement that describes the sources of funding for the work included in this submission. Review the submission guidelines for detailed requirements. View published research articles from *PLOS Neglected Tropical Diseases* for specific examples.<br><br>This statement is required for submission	Research reported in this publication was supported by the National Institute of Allergy and Infectious Diseases of the National Institutes of Health under Award Number U01AI136033.

and **will appear in the published article** if the submission is accepted. Please make sure it is accurate.<br><br>**Funded studies**<br>Enter a statement with the following details:<br>• Initials of the authors who received each award<br>• Grant numbers awarded to each author<br>• The full name of each funder<br>• URL of each funder website<br>• Did the sponsors or funders play any role in the study design, data collection and analysis, decision to publish, or preparation of the manuscript?<br><br>Did you receive funding for this work?"	
Please select the country of your main research funder (please select carefully as in some cases this is used in fee calculation).<br>as follow-up to "**Financial Disclosure**<br><br>Enter a financial disclosure statement that describes the sources of funding for the work included in this submission. Review the submission guidelines for detailed requirements. View published research articles from *PLOS Neglected Tropical Diseases* for specific examples.<br><br>This statement is required for submission and **will appear in the published article** if the submission is accepted. Please make sure it is accurate.<br><br>**Funded studies**<br>Enter a statement with the following details:<br>• Initials of the authors who received each award<br>• Grant numbers awarded to each author<br>• The full name of each funder<br>• URL of each funder website<br>• Did the sponsors or funders play any role in the study design, data collection and analysis, decision to publish, or preparation of the manuscript?	SRI LANKA - LK

Did you receive funding for this work?"	
**Competing Interests**<br><br>On behalf of all authors, disclose any competing interests that could be perceived to bias this work.<br><br>This statement will be typeset if the manuscript is accepted for publication.<br><br>Review the instructions link below and PLOS NTDs' competing interests policy to determine what information must be disclosed at submission.	No competing interests.
**Data Availability**<br><br>Provide a **Data Availability Statement** in the box below. This statement should detail where the data used in this submission can be accessed. This statement will be typeset if the manuscript is accepted for publication.<br><br>Before publication, authors are required to make all data underlying their findings fully available, without restriction. Review our PLOS Data Policy page for detailed information on this policy. Instructions for writing your Data Availability statement can be accessed via the Instructions link below.	All relevant data are within the manuscript and its Supporting Information files.

Manuscript
Click here to access/download;Manuscript;Manuscript_Text with Tab_FIg Final.docx

Title Page

Impact of climatic factors on the temporal variability of sand fly abundance in Sri Lanka: A 2-year longitudinal study

Short title

Climate variability and sand fly abundance in Sri Lanka

Sanath C Senanayake[a#], Prasad Liyanage[b#], Dulani R.K. Pathirage[a], M.F. Raushan Siraj[a], B. G. D. Nissanka Kolitha De Silva[c], Nadira D Karunaweera[a*]

[a]Department of Parasitology, Faculty of Medicine, University of Colombo, Colombo, Sri Lanka.

[b]Department of Research and Evaluation, National Institute of Health Sciences Kalutara, Sri Lanka.

[c]Center for Biotechnology, Department of Zoology, Faculty of Applied Sciences, University of Sri Jayewardenepura, Nugegoda, Sri Lanka.

#Joint first authors

*Corresponding author

Emails:

SCS: sanath@parasit.cmb.ac.lk

PL: prasadgliyanage@gmail.com

DRKP: pathiragedulani@gmail.com

MFRS: raushansiraj@hotmail.com

BGDNK: nissankakolitha@gmail.com

NDK: nadira@parasit.cmb.ac.lk

Abstract

Background

Phlebotomine sandflies are the vectors of leishmaniasis. The sand fly abundance tends to be influenced by context-specific climatic and non-climatic factors. Thus, we aimed to understand how these factors drive sand fly density in ten sentinel sites across Sri Lanka.

Methodology/Principal Findings

We analysed monthly collections of sand flies and climate data from ten sentinel sites representing all geo-climatic zones across Sri Lanka, over 24 months. Site-specific non-climate data was also recorded. The influence of climate and non-climate drivers on sand fly abundance in each site was calculated using distributed lag non-linear models and machine learning, We found that climate plays a major role on sandfly abundance compared to non-climate factors. Increase in rainfall and relative humidity at real time, and ambient temperature and soil temperature with a 2-month lag were associated with a statistically significant increase in sand fly density. The maximum relative risk (RR) was 3.76 (95% CI: 1.58-8.96) for rainfall at 120 mm/month, 2.14 (95% CI: 1.04-4.38) for relative humidity at 82%, 2.81 (95% CI: 1.09-7.35) both at real time. For ambient temperature at 34.5°C, and 11.6 (95%CI; 4.38-30.76) for soil temperature at 31.5ºC; latter 2 variables with a 2-month lag period. A similar delayed association was also seen with the rise of ~~soil temperature and~~ evaporation rates. The real-time increase in ambient temperature, sunshine hours, and evaporation rate, however, reduced sand fly burden homogeneously in all study settings. The high density of chena and coconut

~~cultivation~~, together with low density of dense forests, homesteads, and low human footprint values, positively influenced sandfly densities.

Conclusions/Significance

The findings would enhance understanding of the dynamic influence of environment on sand flies and leishmaniasis spread, laying a foundation for ~~for~~ forecasting of sand fly ~~burden and targeted~~ site-specific interventions for mitigating the growing burden of leishmaniasis, particularly in an era of climate change.

Author Summary

Leishmaniasis, a public health problem in the tropics ~~is transmitted by sand flies~~. Both climatic and non-climatic factors may affect ~~sand flies~~. Thus, we aimed to understand how these factors influence sand fly density in 10 field sites across Sri Lanka with varying eco-climatic conditions.

Monthly collections of sand flies over 24 months were analysed, and the influence of climate and non-climate divers on the sand fly ~~burden~~ was calculated. We found that climate plays a major role on sandfly abundance compared to non-climate factors. An increase in rainfall and relative humidity were associated with a prominent increase in sand fly density. Similar effects were seen with the rise of ambient and soil temperature and evaporation rates, albeit with a 2-month lag period. The increase in ambient temperature, sunshine hours, and evaporation rate in the real-time, however, uniformly reduced sand fly burden. ~~A high~~ chena and coconut ~~cultivation densities~~, along with sparse forests, homesteads, and reduced human footprint indices, positively influenced sandfly densities.

The findings promote a better understanding of the changing climatic and environmental influence on sand fly vectors and leishmaniasis spread, providing a foundation for the development of targeted interventions for sand fly and disease control.

Keywords: *P. argentipes,* sand flies, vector, climate, *Leishmania*, parasite

Introduction

The subfamily Phlebotomine (sand flies) includes as many as 800 species[1]. Sand flies are small (2 to 3mm in size) hairy hematophagous insects that live in warm tropical and sub-tropical regions between 50ºN and 40ºS [2]. Sand flies can transmit several bacterial, viral, and parasitic diseases, including leishmaniasis [3]. Leishmaniases are a group of diseases caused by more than 20 *Leishmania* species of parasites transmitted through the bites of infected female phlebotomine sand flies [1]. More than 90 sand fly vectors are known to transmit the parasite. The type of resultant disease in leishmaniases depends on the causative *Leishmania* species, in large part with clinical manifestations ranging from self-limiting cutaneous lesions to life-threatening visceral disease [1]. The clinical outcome depends on the fine interplay between parasite, vector, and host factors, mainly with the involvement of the immune system [4]. Accordingly, the disease has three main forms; visceral (VL), the most serious form; mucocutaneous (MCL), the most disabling and cutaneous (CL), the most common [1]. It is estimated that between 0.7 to 1 million new cases of cutaneous leishmaniasis occur annually, ranking it third among neglected tropical diseases [5]. Although the disease is endemic in approximately a hundred tropical and sub-tropical countries, over 85% of new cases are concentrated in ten countries: Afghanistan, Algeria, Brazil, Colombia, Iraq, Libya, Pakistan, Peru, the Syrian Arab Republic and Tunisia [1]. The disease is associated with poverty, poor living conditions, and environmental changes such as deforestation, dam construction, irrigation schemes, and urbanization [6–8].

Leishmaniasis is a climate-sensitive disease since the *Phlebotomus* vectors are thermophilic, requiring warm temperatures for survival. The developmental stages of these vectors include eggs, larvae, pupae, and adults. The immature stages do not require standing water to complete the life cycle. The hatching of eggs is highly dependent on temperature, with first instar larvae emerging 12 to 19 days after oviposition, pupae in 25 to 59 days, and adults in 35 to 69 days [9]. Laboratory studies have shown that extreme temperatures below 15°C and above 32°C have a negative impact on the fecundity and longevity of these flies [10]. The influence of weather variables such as rainfall, relative humidity, soil water stress, evaporation rate, wind speed and El Nino Southern Oscillation on the transmission of leishmaniasis had been evaluated in the past across different endemic settings, but the reported associations are inconsistent [11–16]. This heterogeneity could be largely due to the type of data and methods used in the analysis, the location-specific influences of the climate on vector bionomics of the sand fly species and the transmission dynamics of the respective disease entities.

Leishmaniasis has become a significant public health issue in Sri Lanka. In contrast to the declining disease trends observed in other Southeast Asian countries, Sri Lanka has been experiencing a steady increase in case numbers of leishmaniasis with an exponential rise in 2018 [17]. Almost all the leishmaniasis clinical cases in Sri Lanka are CL caused by *Leishmania donovani* [18]. The parasite is probably transmitted through the species *Phlebotomus argentipes glaucus*, which demonstrates zoophilic behavior compared to other related species in India [19,20]. The continuous upsurge of disease transmission in the country warrants urgent attention to design effective control interventions that might enable meeting equivalent elimination targets as established for

VL in the region. These targets involve reducing the incidence to less than one case per 10,000 population [21,22]; the targets specified by the WHO roadmap for neglected tropical diseases 2021-2030 [23]. Climate change and related environmental and socio-economic impacts may catalyze the transmission dynamics in future, further aggravating the existing disease burden. Within this context, it is important to understand the intricate relationship between climate, environmental factors, and sand fly densities to face the growing burden of sand fly-borne diseases. The current study describes the distribution of the sand fly species in different geographic zones related to disease hotspots, and the influence of local weather and non-climate factors on the sand fly abundance in Sri Lanka that are relevant and applicable for the planning of successful interventions for control of leishmaniasis in any endemic country.

Methods

Study areas meteorological and Georeferenced land-use data

Sri Lanka is an island with an area of 65,525 km^2 located between latitudes $5^0$55’ and $9^0$51’N and longitudes $79^0$41 and $81^0$53’E. The country is divided into four climatic zones based predominantly on the rainfall, viz. wet zone, intermediate zone, dry zone, and semi-arid zone. The wet zone, located in the southwest part of the island and central hills, receives the maximum rainfall in the country with an annual average of over 2500mm. The maximum rainfall occurs during the southwest (SW) monsoon from May to September and the northeast (NE) monsoon from November to January. The dry zone covers most parts of the country and receives an annual rainfall between 1200 and 1900mm during the NE monsoon with little or no rain for the rest of the year. An

intermediate zone situated between wet and dry zones in the island receives an average annual rainfall of 1500-2500mm, whereas the semi-arid zones situated within the dry zone of the country receive an average annual rainfall of 800-1200mm [24,25]. The country is divided into 25 districts for administrative purposes, and they are nested within 9 provinces. Nine sentinel sites were strategically chosen to conduct sand fly collections, aiming to closely represent each province and encompass all climate zones. An additional sentinel site, Delft, situated on Delft Island in the Palk Strait, was chosen from the Northern province. The location of the sentinel site within each province was based on the case records of each Medical Officer of Health (MOH) area during the year 2017 as maintained at the Epidemiology Unit, Ministry of Health and also in consultation with the respective Public Health Officials. A perimeter of 5km from the sentinel site was used to study topological factors such as vegetation cover and land use patterns, including water bodies. We also considered the human pressure on the study settings as quantified by the Human Footprint Index (HFI). The ten sentinel sites represented all climate zones of Sri Lanka and were named as per the township that they belonged to, viz. Delft Island, Welioya, Thalawa, Mahaoya, Peradeniya, Ambanpola, Kataragama, Mamadala, Mirigama and Dickwella (Table 1).

Table 1. Characteristics of the sentinel sites.

Province	District	Township and GPS coordinate of site	Altitude category#	Climatic zone##	CL cases reported*
North-western	Kurunegala	Ambanpola 80.2463E/7.89703N	Lowland	Intermediate	Yes
Southern	Matara	Dickwella 80.7015E/5.97627N	Coastal	Wet zone	Yes
Northern	Jaffna	Delft island 79.4314E/9.31090N	Coastal	Dry zone	Rare
Northern	Mullaitivu	Welioya 80.8110E/8.98232N	Coastal	Dry zone	Rare
Southern	Hambantota	Mamadala 80.9667E/6.17158N	Lowland	Dry Zone	Yes
Uva	Monaragala	Kataragama 81.3132E/6.42649N	Lowland	Dry zone	Rare
Eastern	Ampara	Mahaoya 81.3234E/7.48443N	Lowland	Dry zone	Rare
Western	Gampaha	Mirigama 80.0967E/7.22750N	Lowland	Wet zone	Rare
Central	Kandy	Peradeniya 80.6007E/7.26643N	Highland	Wet zone	Rare
North Central	Anuradhapura	Thalawa 80.3400E/8.19928N	Lowland	Dry zone	Yes

#Altitude category: Costal: Surrounds the island with elevation of about 30 m above sea level; Lowland: 30 to 1000m above sea level; Highland: mountainous areas with an elevation of 1000 to 2500 m above sea level.

##Climatic zone: Arbitrary division of the island based on annual rainfall

*The CL cases were considered rare if the annual case incidence was less than 10 cases per 100,000 population in 2017 as per patient data maintained at the Epidemiology Unit, Ministry of Health.

Sand fly collection

The adult sand flies were collected from March 2018 to February 2020 in ten sentinel sites over twenty-four months. Each site was equipped with UV LED CDC light traps (LT) a product of BioQuip, USA (S1 Fig 1A) and cattle-baited net traps (CBNT) (S1 Fig 1B). The CBNTs used were 10 x 10 feet in size and a single animal was placed within the trap and kept overnight. Sand fly samples were collected using a manual aspirator at 10 pm and 4 am. The trapping was conducted for two consecutive days per month using ten LTs and one CBNT per night. However, in Delft Island, sand fly collections were done only twice a year due to logistical constraints. The CBNT was placed in a constant place while the ten LTs were placed in 20 houses in rotation within a radius of 500m to CBNT. A minimum distance of about 50m was maintained between CBNT and LTs. The distance between the houses ranged from 10m to 200m depending on the area. The S1 Fig 2 shows the placement of CBNT and LTs in the Kataragama sentinel site. The same methodology was in all sites including the Delft Island. However, the data from Delft collections were used only for the descriptive analysis and excluded from the time series analysis due to less frequent sampling. Another exception was Peradeniya, where the trapping was done monthly with predominant use of LTs, due to the difficulties in obtaining cattle for CBNTs (only two CBNT cycles were completed). The collected sand flies were preserved in absolute ethanol and transported to the laboratory for further analysis. Species identification of collected sand flies was done based on morphological features using standard keys [26]. Forty-eight cattle-baited trap nights and 960 light trap nights were used across the country to collect *P. argentipes* during the study period. Monthly total (LT total and CBNT total) and average (LT average and CBNT average) *P.*

argentipes sand fly densities were calculated using the insect collections in ten LTs and one CBNT for each site respectively.

Climate data

Monthly mean rainfall, ambient temperature (minimum and maximum), relative humidity, wind speed, soil temperature (measured at 08:30 and 15:30 hours at 5cm and 10cm depth), evaporation and sunshine hours data from March 2018 to February 2020 were obtained from the Meteorological Department of Sri Lanka. The meteorological stations located closest to the sampling sites were selected based on GPS coordinates. We further utilized the remotely sensed climate data downloaded from the ERA5-Land hourly data repository accessible from the Copernicus Climate Change Service Climate Data Store [27]. ~~Remotely sensed~~ climate data for rainfall, temperature, wind speed and soil temperature within a 5km buffer around the geolocations of the surveillance sites were used to supplement the ground level monitoring data where necessary. The ERA datasets (ERA5 and ERA-Interim) do not directly archive Relative Humidity (RH). Therefore, RH was derived from near-surface temperature and dew point temperature based on the Bolton formula [28]. Nevertheless, information on sunshine hours was only accessible for five study locations (Embilipitiya, Thalawa, Ambanpola, Kataragama, and Dickwella). The S1 Fig 3 shows the ~~month specific variability~~ of climate variables averaged across all study settings.

Non-climate contextual information in study settings

Location-specific characteristics, which could further modify the relationship between weather variability and sand fly density, were obtained for the 5km buffer area

around the surveillance sites. Geo-referenced land-use data was obtained from the Sri Lanka Survey Department [29]. The land-use data was clipped and extracted from the 5km buffer around the sentinel site using ArcGIS software. The area of each land-use type was derived using a geometry calculator. The land use values were exported as a database file, which was opened through the Excel application, and the spread of equal land-use categories were totalled using the PivotTable in the Excel application. Land use variables included land areas of paddy fields, dense forests, coconut cultivars, chena cultivars, marshy lands, scrubs lands, rocks, reservoirs, streams, water bodies, cemeteries and homesteads. In addition to these variables, we utilized the human footprint index (HFI), which integrates eight key indicators at a fine spatial resolution (30 arcsec), including built environments, population density, electric infrastructure, crop and pasture lands, roads, railways, and navigable waterways to quantify anthropogenic pressures across nine surveillance sites. The HFI is a dimensionless index calculated as a continuous scale of increasing human pressure from 0 to 50 where more than 12 is considered to be areas with intense human pressure [30]. Furthermore, the HFI provides spatially explicit and temporarily inter-comparable measures of human interaction with the environment and local natural systems. We utilized the most updated HFI maps available, which were generated up to 2019 using a machine-learning method based on the original HFI dataset accessible from 2000 to 2013 [31]. We extracted the average HFI for each study year for a buffer of 5km at each surveillance site. The distribution of these variables among each surveillance site is given in the S1 Table 1.

Leishmaniasis incidence

Leishmaniasis is included in the list of notifiable diseases in Sri Lanka and subjected to mandatory notification to the national integrated communicable disease surveillance system in the country. The number of leishmaniasis cases from March 2017 to February 2020 and the annual average incidence rates of leishmaniasis per 100,000 population by each district were obtained from the Epidemiology Unit, Ministry of Health of Sri Lanka [17].

Statistical analysis

Here we used a combination of two analytical approaches. Firstly, we utilized distributed lag non-linear models (DLNMs) [32] in a two-staged hierarchical meta-analytical framework [33] to assess the delayed (lagged) association between climate variables and sand fly densities across all sentinel sites in Sri Lanka. Secondly, we employed XGBoost, an ensemble ~~decision~~ tree method, to ascertain how these lagged climate variables, along with context-specific non-climate variables, contribute to sand fly densities across study settings [34]. One of the notable advantages of XGBoost over other machine learning algorithms is its capability to adjust for features with minimal data pre-processing and feature engineering requirements. Furthermore, it effectively handles highly nonlinear, correlated, and interactive covariates which cannot be implemented withing the DLNM framework alone.

Evaluating the lagged influence of climate variables on sand fly densities

The DLNMs implemented in the R package *dlnm* (version number 2.4.6) ~~use~~ the concept of creating flexible cross-basis function estimators to capture simultaneously the

delayed and non-linear dependencies of the exposure and outcome data [35]. In the first stage, the exposure-lag-response association for each study setting were flexibly estimated using ground level and remotely sensed weather data. A quasi-Poisson time series regression model was used to account for the over-dispersion of data and the influence of time-varying confounders. The common formula for the first stage sentinel site-specific models for weather variables and sand fly density indices is given as

$$VI\ i\sim quasiPoisson(\mu t)$$

$$E(VI_{ti}) = \beta i+f(Weathre_{ti},vardf,lagdf) + s(T_{ti},timedf)$$

Where $E(VI_{ti})$ was the expected value for each sand fly density measurements (LT average, LT total and CBNT average) obtained by LTs and CBNTs in each month ($_t$) in each surveillance setting ($_i$). β was the intercept, and_$f\ (Weathre_{ti},vardf,lagdf)$ was the cross-basis function for each weather variable (rainfall, maximum, minimum and mean temperature, soil temperature, relative humidity, sunshine hours etc. respectively, in each model). The *vardf* and *lagdf* were the corresponding degrees of freedom set for weather variables. $s\ (T_{ti},timedf)$ was the smooth function of time with the degrees of freedom used to account for the time-varying confounders on the outcome. We used lag up to three months considering the lifecycle of sandfly vectors to capture all biologically plausible associations between climate variables and sand fly densities.

In the second stage, the surveillance site-specific exposure-response associations were meta-analysed to obtain joint estimates for the country accounting for within and in-

between surveillance site-level variability. The model output was given as a relative risk (RR) estimate calculated for the full range of exposure values with reference to a risk at a predetermined central reference. We used a multivariate extension to the Cochrane Q-test of heterogeneity to assess the statistical significance of the heterogeneity of the estimates at each study setting and it was further quantified by using I^2 statistics [36]. The models were evaluated using the quasi-Akaike information criterion (q-AIC) [37]. q-AIC values derived during the model building and selection procedure for each sandfly density measurement are given in the S1 Table 2. The lowest q-AIC values observed for LT average indicate the better model fit compared to CBNT and LT monthly total for all weather parameters. Therefore, we selected sand fly densities obtained using LT for our primary analysis and the results were compared with CBNT where relevant. Definition of the cross-basis functions with respect to different knot positioning for the best-fit models are reported in the S1 Table 3. We used *mvmeta* package (version number 1.0.3) for the second stage multi-variate meta-analysis [33,38]. The divisional heterogeneity of each climate variable is presented in the S1 Table 4.

Evaluating the relative contribution of climate and non-climate variables on sand fly densities

XGBoost, that uses gradient boosting, has a comparative advantage over other tree-boosting methods in terms of its versatility, scalability, speed, and optimization to solve complex problems [35]. Recent advancements in machine-learning have led to the development of explanatory frameworks for interpreting the model outputs. These are often referred to as explainable AI (XAI). We coupled the XGBoost output with the XAI

post-processed model interpretation framework, Shapley Additive Explanation (SHAP), which allows us to rank the features of the model (climate and non-climate variables in the present setting) in their order of contribution [39]. SHAP determines the importance of the feature by comparing a model's predictions with and without a specific feature, considering all possible feature combinations for each observation. The ranking of features is based on their individual contributions for each observation and then averaged across all observations.

All lagged climate variables identified using the DLNM approach described above, along with non-climate variables given in the S1 Table 1, were incorporated in the XGBoost model. First, we trained the model using XGboost gradient-boosted tree regression algorithm using all 23 variables. To maximize the model’s performance, we used a random search algorithm to tune hyperparameters. Specifically, we tuned *max_depth,* which defines the maximum depth of a tree, *eta,* step size shrinkage parameter to prevent overfitting*, subsample,* a subsample ratio of the training instances*, colsample_bytree,* a subsample ratio of columns for each tree*,* and *min_child_weight*, a minimum number of instances needed to be in each tree node. Details regarding the hyperparameter settings and final optimal parameters can be found in S1 Table 5. We also used the 5-fold cross-validation to ensure the model is not an overfit to the data. The model’s performance was assessed using R-squared values. The model fit was further validated using Adj-R squared and RMSE metrics through a secondary analysis involving random partitioning of the data into training (80%) and test (20%) sets. We then applied SHAP on the best-fit model to rank the features in the order of their contribution. SHAP values for each variable were computed to evaluate their positive and negative impacts on sand fly vector

densities and presented in a global feature importance bar diagram and local explanation summary plots. All analytical steps were implemented within the R statistical environment (version 4.1.0) [40].

Results

Sand fly species composition and sex ratio

P. argentipes was the predominant sand fly species captured, accounting for 38,594 sand flies (female: male ratio = 4,246:34,348), (Table 2). The remaining sand flies (n=333; <1%) belonged to the genus *Sergentomyia* (data not shown). The female-to-male ratio in the total *P.argentipes* sand fly collection was approximately 1:8.2, indicating that there were approximately eight times more males than females in the collection. The female-to-male ratio of *P. argentipes* varied depending on the trap type, with a ratio of 1:9.9 in the cattle-baited traps and 1:1.8 in the light traps.

Table 2: The total of *P. argentipes* recorded monthly using different collection methods and annual average density of *P. argentipes* in sentinel sites from March 2018 Feb 2020.

Surveillance Site	CBNT	LT	Total	Annual average density of *P.argentipes* sandflies
Ambanpola (NW)	4619	358	4977	2488.5
Dickwella (SP)	7720	353	8073	4036.5
Delft (NP)	1917	29	1946*	5838*
Mamadala (SP)	8704	387	9091	4545.5
Kataragama (UP)	3825	305	4130	2065.0

Mahaoya (EP)	1859	226	2085	1042.5
Mirigama (WP)	3016	171	3187	1593.5
Peradeniya (CP)	72	166	238**	515**
Talawa (NC)	2473	236	2709	1354.5
Welioya (NP)	1691	467	2158	1079
**TOTAL**	35896<br>(♀=3290)<br>♀:♂= 1:9.9	2698<br>(♀=956)<br>♀:♂= 1:1.8	38594<br>(♀=4,246)<br>♀:♂= 1:8.2	

*Based on 4 collections during the period
** 2 CBNT and 24 LT cycles collections

Spatial ~~dynamics~~

The spatial densities of *P. argentipes* captured were highly heterogeneous and variable. Based on the density the sites were arbitrarily classified into High >2500, Mid 1500-2500 and Low <1500 zones. Mamadala, Delft Island and Dickwella were within the high sand fly density zone, whereas Kataragama, Mirigama and Ambanpola were in mid sand fly density zone, and Thalawa, Welioya, Peradeniya and Mahaoya were within the low sand fly zone (Table 2). The sand fly densities positively correlated with the leishmaniasis incidence in these areas with a tendency for high disease burden areas to record high sand fly densities (Fig 1) though this pattern was not consistent in all districts with the Spearman's rank correlation coefficient being 0.57, (p-value > 0.088).

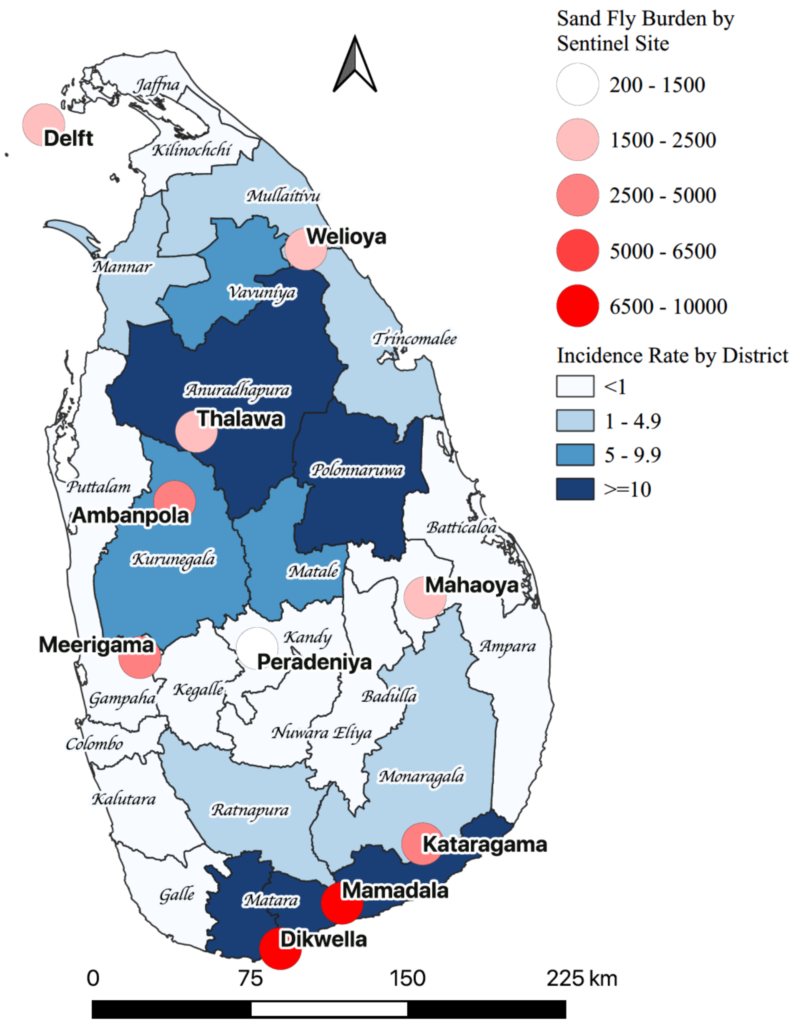

Fig 1. Sand fly burden by sentinel site and annual average leishmaniasis incidence rate (per 1000 population) by district from 2018 to 2020 in Sri Lanka. The blue shaded areas indicate the case burden and the red shaded circles show the geographical distribution and the cumulative number of sand flies collected at each sentinel site for the study period. Black solid lines in the map represent the boundaries of administrative districts. Source of the base file: https://data.humdata.org/dataset/sri-lanka-administrative-levels-0-4-boundaries

Exposure-lag-response associations between weather variables and leishmaniasis vector indices

The overall pooled results of the two staged hierarchical meta-analysis using DLNM approach suggested that rainfall, ambient temperature, soil temperature measured at 10cm depth at 8.30 am, sunshine hours, mean relative humidity, wind speed and evaporation were associated with leishmaniasis vector activity (as measured by the UV LED CDC traps) at different lag dimensions across all study settings. The exposure-response curves of these climate variables with the corresponding statistically significant lags are given in Fig 2 and Fig 3. The full spectrum of the associations (lag 0 to lag 3) of each climate variable are given in the S1 Fig 4 to Fig 10.

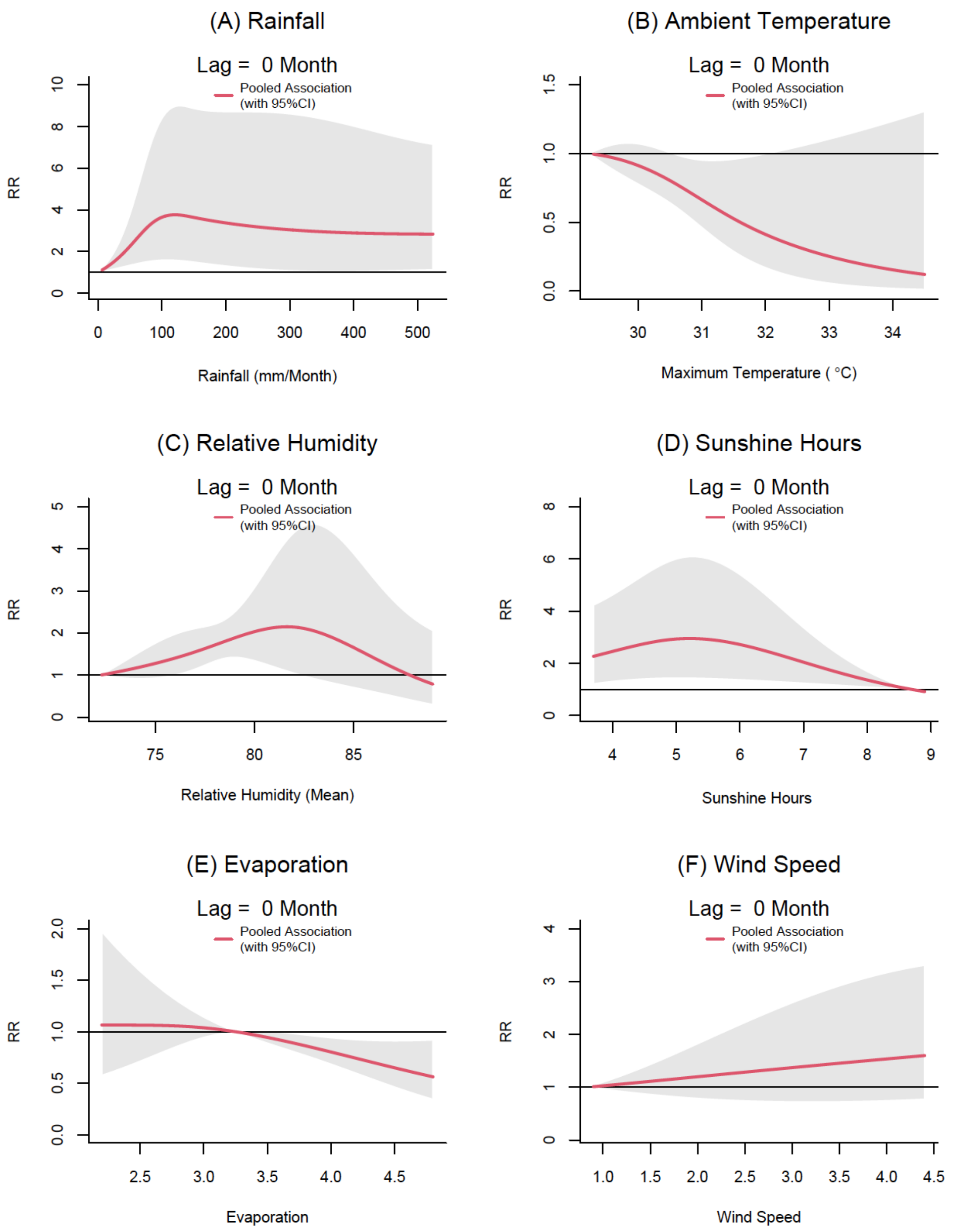

Fig 2. Relative risk (RR) of leishmaniasis vector activity (measured by UV LED CDC traps) by rainfall (A), ambient temperature (maximum temperature) (B), average relative humidity (C), sunshine hours (D), evaporation (E) and wind speed (F) at a lag of 0 months. The exposure-response functions at lag of 0 month were predicted from

the pooled exposure-response function obtained from the meta-analysis for all surveillance sites in Sri Lanka, 2018–20. Shaded areas are 95% CIs. Relative risks were calculated with reference to the risk at a rainfall value of 0 mm per month, maximum temperature of 29.3^{0}C, average relative humidity of 72.25, average evaporation of 3.3mm and wind speed of kmh^{-1}. The most important lags for each exposure variable were selected for presentation. The full spectrum of exposure-lag response associations is given in S1, Fig 4 to Fig 10.

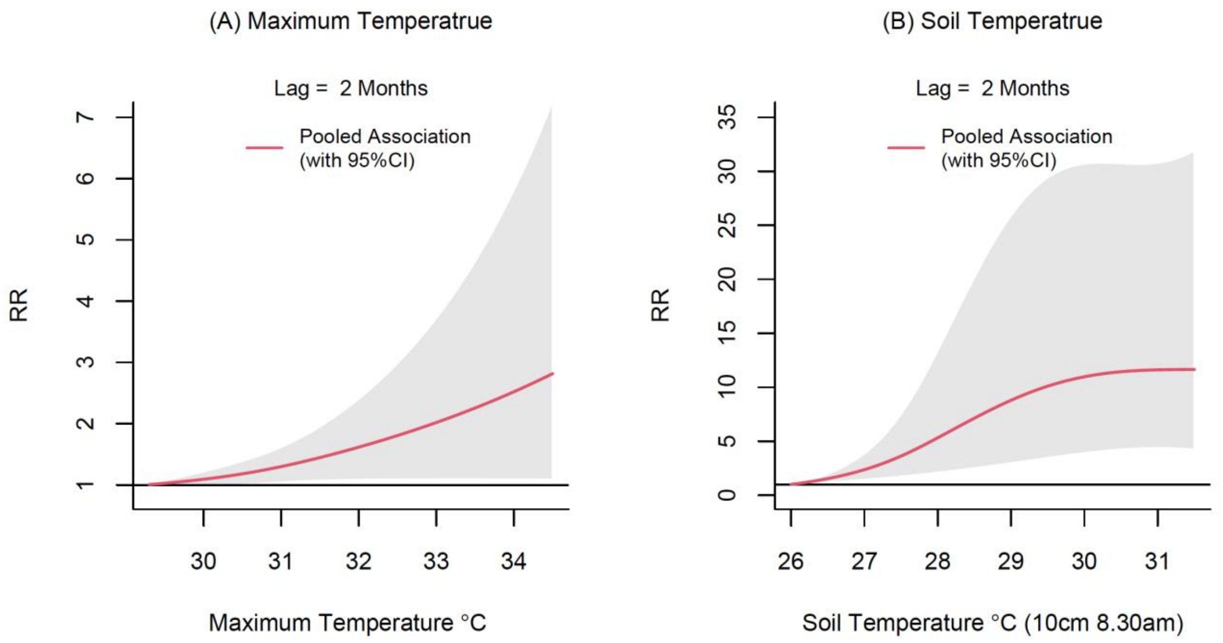

Fig 3. Relative risk (RR) of leishmaniasis vector activity (measured by UV LED CDC traps) by ambient temperature (maximum) (panel A) and soil temperature measured at 8.30 am at 10cm below the surface and evaporation (panel B) at the lag of 2 months. The exposure-response functions at each lag were predicted from the pooled exposure-response function obtained from the meta-analysis for all surveillance sites in Sri Lanka, 2018–20. Shaded areas are 95% CIs. Relative risks were calculated with

reference to the risk at a soil temperature of 26^{0}C. The full spectrum of the associations is given in S1, Fig 5 and Fig 9.

Relative contribution of climate and non-climate variables on sand fly densities

Fig 4 ranks the twenty predictor variables (climate and non-climate) based on their SHAP values in descending order. These values elucidate the significance of each variable in influencing sand fly densities, as measured by the light trap (LT per trap) across all surveillance sites. The global feature importance plot illustrates the relative contribution of each feature, while the local explanation summary demonstrates how these features impact sand fly density across the entire spectrum of values.

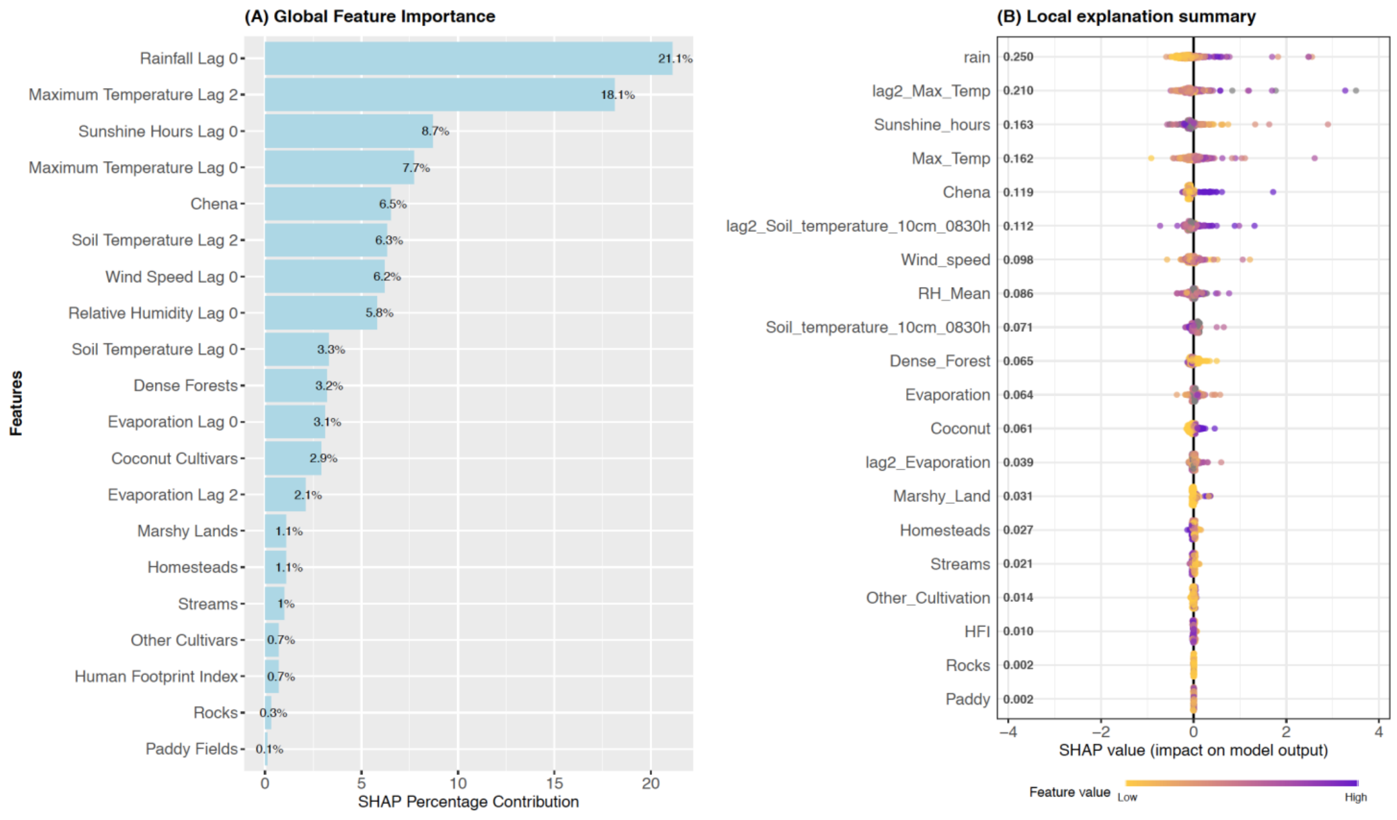

Fig 4. SHAP feature importance plot for sand fly densities measured by light traps. The panel A (Global Feature of Importance) bar chart presents the percentage contribution of mean SHAP values estimated for each feature. The panel B (Local Explanation Summary) is a set of beeswarm plots showing feature impact on the model output in their full range of values. The purple color indicates a higher value of corresponding variables and the yellow color indicates a lower value. The dot's position on the x-axis shows the impact that feature on the model's prediction of sand fly densities. SHAP dependency plot for each variable with better visual effect is given in the S1 Figs 11 and 12.

Climate variables appeared to be relatively more important for the sand fly densities when compared to the non-climate variables. Rainfall showed the highest contribution, followed by maximum temperature lag 2, sunshine hours lag 0, maximum temperature lag 0, soil temperature lag 2, wind speed lag 0, relative humidity lag 0 and evaporation lag 0. Out of the non-climate contextual factors, chena cultivation and dense forest were relatively important compared to other non-climate variables.

Rainfall

When pooled across all the sentinel sites, the rainfall appeared to be associated with the risk of increasing sand fly density measured by UV LED CDC trap at lag of 0 (Fig 2 panel A). As shown in the S1 Fig 4, when the lags are increasing, the exposure-response associations become less obvious. With reference to the risk at a rainfall value of 0, the increase in rainfall was associated with the statistically significant increase in the

RR of sand fly density throughout its range of values. The maximum RR of 3.76 with a 95% CI of 1.58 to 8.96 was observed at the rainfall of 120 mm per month. Thereafter, the RR was observed to slightly decrease with increasing rainfall up to the extreme rainfall value of 524mm per month (RR of 2.83 with 95% CI of 1.12 to 7.14). However, a statistically significant Q test of heterogeneity (p-value 0.003) revealed a substantial variation in the exposure-response association among surveillance sites, with an I statistic of 49%. The local explanation summary (Fig 4, panel B) and SHAP dependence plots (S1 Fig 11) demonstrate that increasing rainfall values predominantly positively influence sand fly densities.

Ambient Temperature

At the lag of 0 months, the increase in ambient temperature (maximum temperature) appeared to reduce the RR of sand fly density (Fig 2 panel B). With reference to the lowest temperature value in the range (29.3^{o}C), the sand fly activity appeared to be reduced by each unit increase in the temperature. The associations were statistically significant between 30.6 ^{o}C to 32 ^{o}C and the minimum relative risk observed at the temperature value of 34.5 ^{o}C was 0.12 (95%CI; 0.01 to 1.3). Conversely, the increasing maximum temperature at a lag of 2 months increased the RR of sand fly density and was more influential compared to the lag 0 effect (Fig 3 panel A and Fig 4). The highest RR observed was 2.81(95% CI; 1.09 to 7.35) at 34.5 ^{o}C. The temperature-sand fly density association was homogeneous across all surveillance sites (S1 Table 4).

Relative humidity

With reference to the minimum RH value of 72.25, the relative risk of vector activity appeared to increase with the increase in RH up to 82 (2.14; 95% CI = 1.04 to 4.38) at the lag of 0 months Fig 2 panel C. The RR however, Q test was statistically significant (p-value 0.012) with I^2 of 49.6% indicating substantial heterogeneity among surveillance sites. The SHAP dependency plot (S1 Fig 11) illustrates that increasing RH elevates the RR, with extreme values tending to decrease it.

Sunshine hours

Increasing sunshine hours appeared to reduce the RR of sand fly densities at a lag of 0 months. The maximum relative risk observed (2.93; 95% CI = 1.43 to 6.0) was at 5 hours of sunshine per day (Fig 2 panel D). When the daily average sunshine hours further increased, the relative risk of vector activity also appeared to decrease. A similar pattern was observed at a lag of 1 month. The association was not statistically significant, with a further increase in lags (S1 Fig 7) The observation was homogeneous across all the settings as suggested by the non-significant Q test (2.77, p-value = 0.950). Our analysis was limited to five surveillance sites (Embilipitiya, Thalawa, Ambanpola, Katharagama and Dikwella) due to the limited availability of ground and remote sensing data on sunshine hours.

Wind speed

An increase in wind speed appeared to increase the risk of vector activity at the lag of 0 months (Fig, 2F). However, the associations were not statistically significant up to a lag of 3 months (S1 Fig, 8). The associations appeared to be homogeneous across sites

(S1 Table 4). The SHAP dependency plot (S1 Fig 11) illustrates that the extreme values of wind speed have a negative influence on sand fly densities.

Soil temperature

Increasing soil temperature with a lag of 2 months and measured at 8.30 am at 10cm below the surface was associated with increasing relative risk of sand fly densities (Fig 3 panel B). The risk of vector activity started to increase at a lag of 1 month and reached its maximum at the 2-month lag period before reducing at the lag of 3 months (S1 Fig 9) At a lag of 2 months, the relative risk of sand fly density peaked at 11.6 (95% CI: 4.38 to 30.76) when the soil temperature reached its maximum value of 31.5°C. Similar to the ambient temperature the relative risk of sand fly activity decreased with increasing soil temperature at a lag of 0 months. With reference to the risk estimated at 26^{0}C, the lowest relative risk was observed to be 0.12 (95%CI; 0.03 to 0.40) at a soil temperature value of 31^{0}C. At a lag of 0 months, the observed reduction in the risk of vector activity was homogeneous across study settings as suggested by the non-significant Q test. The heterogeneity was statistically significant at a lag of 2 months (S1 Table 4).

Evaporation

With reference to the evaporation value of 3.25 (which was the median evaporation value observed when averaged across all the settings) the risk of sand fly density appeared to decrease with increasing evaporation at a lag of 0 months when the evaporation value exceeded 3.6 (S1 Fig 10). The minimum relative risk observed (0.56; 95% CI = 0.92 to 0.34) was at an evaporation value of 4.8. An opposite pattern was observed at a lag of 2

months where the RR appeared to increase with increasing evaporation (S1 Fig10). The association was not statistically significant with a further increase in lag periods. The observation was homogeneous across all the settings as suggested by the Q test (S1 Table 3, p-value = 0.339). Evaporation appeared to be the least influential climate variable based on the SHAP ranking (Fig 4).

Non-climate contextual variables

The high land area of chena cultivation, low land areas of dense forests and high land areas of coconut cultivation emerged as important non-climatic factors influencing sand fly densities across the surveillance sites. Moreover, other cultivars, marshy lands and paddy fields in comparatively large land areas exhibited a positive influence on sand fly densities. Conversely, a high density of homesteads and high values of the human footprint index were associated with decreased sand fly densities. Additionally, large land areas with streams were found to have a diminishing effect on sand fly density (Fig 4 and S1 Fig 12).

Discussion

The aim of the study was to describe the distribution of the sand fly species in different geographic zones related to disease hotspots, and quantify the effect of climatic and non-climate variables on sand fly vector abundance in selected sentinel sites that represent the varying geographical and climatic zones in Sri Lanka. The temporal variability of sand fly densities was investigated over a period of 24 months through a uniform trap placement across the surveillance sites. Concurrent weather variables viz. monthly

average rainfall, ambient temperature, relative humidity, wind speed, soil temperature, evaporation and sunshine hours and non-climate contextual information collected in proximity to the surveillance site were used to quantify their location specific influence on the sand fly densities. Using a combination of statistical modeling and a machine learning approach we were able to identify climate and non-climate drivers of sand fly vector abundance and their relative importance across Sri Lanka.

The high attractiveness of sand flies to cattle as demonstrated by high counts in CBNTs may be attributed to their preference for animal blood, which is enhanced by its greater body size and CO_2/odour output, and the availability of the cattle for a sustained and successful blood feed [41,42]. The densities of sand flies appeared to differ based on the climatic zone in which the sentinel sites were located. Among the study sites, Kataragama, Mamadala, and Dickwella (dry climatic zone) exhibited higher densities of *P. argentipes*. In contrast, Ambanpola and Mahaoya (intermediate zone) and Welioya (in the dry zone) had lower sand fly collections. Previous studies conducted in Sri Lanka have also reported *P. argentipes* as the predominant species of sand flies [43–45]. However, the current study demonstrates, for the first time, the widespread presence of *P. argentipes* across the country, including Delft Island. The sex ratio of sand flies collected in this study was significantly biased towards males in the genus *Phlebotomus*. This is a known phenomenon where male flies are attracted in large numbers to traps containing female flies [46].

A positive but not statistically significant correlation was observed between sand fly density and the incidence of leishmaniasis cases recorded from 2018 to 2020. However, this finding may not be surprising since leishmaniasis is a chronic disease, and the

manifestation of symptoms typically occurs months or even years after exposure. Additionally, there are multiple factors affecting the transmission of infection, with vector abundance being one among many such variables [47].

Our analysis revealed that climate conditions conducive to sand fly activity are characterised by a combination of moderate rainfall, low sunshine hours, low ambient temperatures, high relative humidity, and low evaporation rates. The combination of above climatic factors creates an environment that supports increasing sand fly activity in real-time and further modified by various non-climatic factors [48]. We noticed a minor decrease in the relative risk (RR) of sandfly activities during periods of extreme rainfall. However, this observation was not consistent across all surveillance sites. Once averaged across all nine study settings, the increasing ambient and soil temperature at real-time (lag zero) negatively correlated with the sand fly activity reducing the relative risk below one. Similarly, laboratory experimental studies have found that increasing temperatures more than 32^{0}C was associated with higher mortality rates (around 72%) of adult sand flies [10]. However, the ambient temperature (maximum) and soil temperature at a lag of two months exhibited a statistically significant association with an increased risk of sand fly vector activity. Remarkably, the ambient temperature with a lag of two months emerged as a highly influential factor, second only to rainfall in its impact on sand fly densities. The studies have found that complete egg to adult development of the sand fly species was temperature-dependent and ranged from 27.89 (+/- 1.88) days at 32°C to 246.43 (+/- 13.83) at 18°C [49]. This time lag between oviposition and emergence of adults correlates with the observed time lag of two months found between the soil temperature and sand fly abundance in all Sri Lankan study settings, which might be well

within the favourable range for egg hatching and larval development. Therefore, it would be reasonable to extrapolate that the exposure to the optimal soil temperatures two months ago may have produced a large number of adults that were attracted to the light traps at the time of surveillance. Increasing mean RH up to 82 during the same month of surveillance may have created a suitable environment for the sand flies to be active. The negative effect of the evaporation and the higher RR observed for the low number of sunshine hours at lag zero signify the relative inactivity of the sand flies during extremely dry conditions with a low RH. Among the factors investigated, wind speed is likely to have the potential to influence the dynamic behaviour of sand flies, particularly in terms of gene flow between populations without geographical barriers. The gene flow can facilitate the transfer of genes that promote sand fly survival, such as insecticide resistance genes [45], which can have negative implications for vector control programs. Although wind speed emerged as one of the influential climate variables, with extremely high values having a negative influence observed in the machine learning approach, our study did not identify a biologically plausible lagged relationship between sand fly density and wind speed.

Among the non-climate variables measured within a five-kilometre radius from the surveillance sites, cultivation lands have emerged as significant factors influencing sand fly vector densities. Notably, chena cultivation, coconut cultivars, and to some extent, paddy cultivars appeared to play important roles. Alongside other cultivars categorized under broader cultivation lands, these agricultural areas are primarily situated in the dry and intermediate zones of the country. The presence of a low volume of dense forests and streams also suggests conditions typical of the dry zone, potentially contributing to

the higher influence on sand fly vector densities observed at the lower end of their range. However, non-agricultural marshy lands, commonly found in the wet and intermediate zones, were also found to have a positive effect. Furthermore, agricultural areas in the dry and intermediate zones in the country typically exhibit lower population densities and reduced human activity compared to urban or residential areas. In our study, we observed that a low number of homesteads and lower values of the Human Footprint Index (HFI) positively influenced sand fly densities. This phenomenon can be attributed to the favourable breeding and resting conditions for sand flies in these less disturbed environments. The lower population density and reduced human activity in agricultural lands may contribute to the proliferation of sand fly populations, ultimately resulting in higher densities observed in these areas. Agricultural practices may create suitable breeding grounds due to the associated high prevalence of rodents, livestock shelters and irrigation canals [48,50]. A positive and favourable interaction of the weather variables in the dry zone may be more conducive for the sand fly vectors to thrive and transmit the *Leishmania* parasites.

The sand fly vector burden varied in relation to selected climatic variables, either at real-time or with a time lag. The findings may be utilized in forecasting vector burden (thus the risk of disease transmission) based on climatic data to facilitate the planning of effective control strategies against leishmaniasis in endemic countries. Further research endeavours aimed at assessing the impact of environmental factors, including wind speed, may provide valuable insights to aid the combat of future public health challenges, particularly those associated with climate change and for the development of location-specific strategic plans for disease control.

Limitations

The data on sunshine hours was limited to five surveillance sites. Furthermore, the density of sand flies in Delft Island was monitored only bi-annually due to logistical constraints and was analysed against the climatic data recorded off the Jaffna peninsula (40 km away from Delft), which has the nearest meteorological station, which is also a limitation of this study. The boundary knots of the cross-basis matrices were positioned at the average values of the maximum and minimum values of all division-specific climate variables. This approach aimed to obtain a meaningful estimate for the second-stage meta-analysis. However, it led to limitations in exposure range by excluding extreme values of the respective variables observed in certain surveillance settings. As a result, the parameter estimates were constrained and unable to capture the full range of exposure in real-world situations. This limitation does not apply to the XGBoost approach, thereby complementing the interpretation constraints of the DLNM approach by capturing the full exposure range. The estimated relative risk values are likely to be context-dependent and may have limitations in terms of generalizability. However, the lagged effect is believed to have universal applicability due to its association with the biologically plausible temporal dynamics of sand fly vector life cycles.

Implications of the findings for vector control, disease control, climate change and meeting WHO 2030 targets for NTDs

The findings are significant in forecasting vector abundance and designing effective strategies to curtail leishmaniasis transmission in a given setting during an era of

escalating concern over climate change. This study while adding to the evidence linking leishmaniasis incidence with changes in environmental factors, provides novel information on the likely effect of selected environmental factors on developing sand fly stages in the soil, with the resultant lag effect observed on adult sand fly abundance. This observation may be used in establishing an early disease warning system for local populations to aid control, which may also be used as a model for other endemic countries. Favourable climatic conditions in terms of temperature, rainfall and humidity experienced by the local sand fly population are likely to promote leishmaniasis transmission. A temperature range between 29.9 and 33.0 ^{o}C, a humidity level up to 82% and the presence of moderate rainfall (up to 120mm per month) were optimal parameters for the development and longevity of sand flies, increasing the risk of transmission of leishmaniasis. Furthermore, the results presented here suggest that the state of vegetation may also play a role in establishing favourable environmental conditions for leishmaniasis across Sri Lanka. Overall, these findings demand a regionally-coordinated strategic plan to address the apparent threat of increasing risk of leishmaniasis, particularly in the face of changing climatic factors and to test the potential use of vector abundance forecasting in planning vector control for better impact. Such an effort may increase the chance of achieving the WHO 2030 targets for effective control and elimination of NTDs in the region.

Conclusions

The sand fly abundance correlates with environmental parameters such as rainfall, soil temperature, ambient temperature, relative humidity, evaporation rate and sunshine

hours either at real-time or with a time lag. The findings can be used for forecasting of sand fly densities and the design of effective strategies for leishmaniasis transmission accordingly in a given setting. Combining these environmental findings with epidemiological and demographic data and robust surveillance systems will be essential to further enhance our ability to predict disease outbreaks. This holistic approach, incorporating a comprehensive understanding of the environmental factors and the ecology of leishmaniasis, will refine existing approaches and develop more accurate disease outbreak predictions to enable effective infection prevention and control.

Additional files

Additional file: S1 Supplement

Acknowledgements

We acknowledge Mr. Sunil Shantha and Mr. M. P. Ariyapala for field assistance to collect sand flies, and the Head and staff of the Parasitic Diseases Research Unit, Department of Parasitology, Faculty of Medicine, University of Colombo for logistical help. Research reported in this publication was supported by the National Institute of Allergy and Infectious Diseases of the National Institutes of Health under Award Number U01AI136033. The content is solely the responsibility of the authors and does not necessarily represent the official views of the National Institutes of Health, USA.

Abbreviations

DLNM: Distributed Lag Non-Linear; VL: Visceral leishmaniasis; MCL: Mucocutaneous leishmaniasis; CL: Cutaneous leishmaniasis; WHO: World Health Organization; RDHS:

Regional Director of Health Services; CBNT: Cattle-baited net trap; UV light trap: Ultraviolet light trap; NE: Northeast; SW: Southwest; LT: Light trap; RR: Relative risk; MOH: Medical Officer of Health; q-AIC: Quasi-Akaike information criterion; CI: Confident Interval; RH: Relative humidity; CO_2 : Carbon dioxide; NTDs: Neglected Tropical Diseases.

Ethics approval and consent to participate

Ethics approval for the study was granted by the Ethics Review Committee, Faculty of Medicine, University of Colombo, Sri Lanka (Ref no. EC-17-062).

Consent for publication

Not applicable.

Availability of data and materials

Climate data collected for the study is available upon request from the Department of Parasitology, Faculty of Medicine, University of Colombo, Colombo, Sri Lanka and Department of Research and Evaluation, National Institute of Health Sciences Kalutara, Sri Lanka.

Competing interests

The authors declare that they have no competing interests.

Funding

This work was supported by the National Institute of Allergy and Infectious Diseases of the National Institutes of Health, USA, under award number U01AI136033. The content is solely the responsibility of the authors and does not necessarily represent the official views of the National Institutes of Health.

Author contributions

SCS: Planned and executed the field studies, designed methodology and collected the data.

PL: Data analysis, application of statistical methods, use of computational methods to analyse study data, drafted sections of the manuscript.

DRKP: Data analysis and initial draft of manuscript

MFRS: Data collection, analysis and initital draft of manuscript

BGDNK: Provided inputs in research design and manuscript editing

NDK: Formulated research aims and ideas, acquired financial support for the project, oversight and leadership responsibility for the study and preparation of the manuscript.

References

1. WHO Report. WHO fact sheet: Leishmaniasis. 2023. Available: https://www.who.int/news-room/fact-sheets/detail/leishmaniasis
2. Alexander B, Maroli M. Control of phlebotomine sandflies. Med Vet Entomol. 2003;17: 1–18. doi:10.1046/j.1365-2915.2003.00420.x
3. Ayhan N, N. Charrel R. Sandfly-Borne Viruses of Demonstrated/Relevant Medical Importance. Vectors and Vector-Borne Zoonotic Diseases. IntechOpen; 2019.

doi:10.5772/intechopen.81023

4. Colmenares M, Kar S, Goldsmith-Pestana K, McMahon-Pratt D. Mechanisms of pathogenesis: Differences amongst Leishmania species. Transactions of the Royal Society of Tropical Medicine and Hygiene. Oxford Academic; 2002. p. S3. doi:10.1016/s0035-9203(02)90044-1
5. Scheufele CJ, Giesey RL, Delost GR. The global, regional, and national burden of leishmaniasis: An ecologic analysis from the Global Burden of Disease Study 1990-2017. Journal of the American Academy of Dermatology. Mosby Inc.; 2021. pp. 1203–1205. doi:10.1016/j.jaad.2020.08.043
6. Savić S, Savić S. Vectors and Vector-Borne Zoonotic Diseases. Vectors and Vector-Borne Zoonotic Diseases. IntechOpen; 2019. doi:10.5772/intechopen.77985
7. ~~Colmenares M, Kar S, Goldsmith-Pestana K, McMahon-Pratt D. Mechanisms of pathogenesis: Differences amongst Leishmania species. Trans R Soc Trop Med Hyg. 2002;96: S3. doi:10.1016/s0035-9203(02)90044-1~~
8. Scheufele CJ, Giesey RL, Delost GR. The global, regional, and national burden of leishmaniasis: An ecologic analysis from the Global Burden of Disease Study 1990-2017. Journal of the American Academy of Dermatology. J Am Acad Dermatol; 2021. pp. 1203–1205. doi:10.1016/j.jaad.2020.08.043
9. Volf P, Volfova V. Establishment and maintenance of sand fly colonies. J Vector Ecol. 2011;36: 1–9. doi:10.1111/j.1948-7134.2011.00106.x
10. Kasap OE, Alten B. Laboratory estimation of degree-day developmental requirements of Phlebotomus papatasi (Diptera: Psychodidae). J Vector Ecol. 2005;30: 328–333.
11. Boussaa S, Kahime K, Samy AM, Salem A Ben, Boumezzough A. Species composition of sand flies and bionomics of Phlebotomus papatasi and P. sergenti (Diptera: Psychodidae) in cutaneous leishmaniasis endemic foci, Morocco. Parasites and Vectors. 2016;9: 1–11. doi:10.1186/s13071-016-1343-6
12. Chaves LF, Calzada JE, Valderrama A, Saldaña A. Cutaneous Leishmaniasis and Sand Fly Fluctuations Are Associated with El Niño in Panamá. PLoS Negl Trop Dis. 2014;8: e3210. doi:10.1371/journal.pntd.0003210

13. De Oliveira EF, Casaril AE, Fernandes WS, De Saboya Ravanelli M, De Medeiros MJ, Gamarra RM, et al. Monthly distribution of phlebotomine sand flies, and biotic and abiotic factors related to their abundance, in an urban area to which visceral leishmaniasis is endemic in Corumbá, Brazil. PLoS One. 2016;11: e0165155. doi:10.1371/journal.pone.0165155

14. Faraj C, Ouahabi S, Adlaoui EB, El Elkohli M, Lakraa L, El Rhazi M, et al. Insecticide susceptibility status of Phlebotomus (Paraphlebotomus) sergenti and Phlebotomus (Phlebotomus) papatasi in endemic foci of cutaneous leishmaniasis in Morocco. Parasites and Vectors. 2012;5: 51. doi:10.1186/1756-3305-5-51

15. Ramezankhani R, Sajjadi N, Nezakati esmaeilzadeh R, Jozi SA, Shirzadi MR. Climate and environmental factors affecting the incidence of cutaneous leishmaniasis in Isfahan, Iran. Environ Sci Pollut Res Int. 2018;25: 11516–11526. doi:10.1007/s11356-018-1340-8

16. Rodgers M de SM, Fonseca E, Nieto P del M, Malone JB, Luvall JC, McCarroll JC, et al. Use of soil moisture active passive satellite data and WorldClim 2.0 data to predict the potential distribution of visceral leishmaniasis and its vector Lutzomyia longipalpis in Sao Paulo and Bahia states, Brazil. Geospat Health. 2022;17. doi:10.4081/gh.2022.1095

17. Karunaweera ND, Senanayake S, Ginige S, Silva H, Manamperi N, Samaranayake N, et al. Spatiotemporal distribution of cutaneous leishmaniasis in Sri Lanka and future case burden estimates. PLoS Negl Trop Dis. 2021;15: e0009346. doi:10.1371/journal.pntd.0009346

18. Karunaweera ND, Pratlong F, Siriwardane HVYD, Ihalamulla RL, Dedet JP. Sri Lankan cutaneous leishmaniasis is caused by Leishmania donovani zymodeme MON-37. Trans R Soc Trop Med Hyg. 2003;97: 380–381. doi:10.1016/S0035-9203(03)90061-7

19. Senanayake SASC, Abeyewicreme W, Dotson EM, Karunaweera ND. Characteristics of phlebotomine sandflies in selected areas of Sri Lanka. Southeast Asian J Trop Med Public Health. 2015;46: 994–1004. Available: /pmc/articles/PMC6206496/

20. Gajapathy K, Peiris LB, Goodacre SL, Silva A, Jude PJ, Surendran SN. Molecular

identification of potential leishmaniasis vector species within the Phlebotomus (Euphlebotomus) argentipes species complex in Sri Lanka. Parasites and Vectors. 2013;6: 1–9. doi:10.1186/1756-3305-6-302

21. Rijal S, Sundar S, Mondal D, Das P, Alvar J, Boelaert M. Eliminating visceral leishmaniasis in South Asia: The road ahead. BMJ Open. British Medical Journal Publishing Group; 2019. doi:10.1136/bmj.k5224

22. WHO EMRO | Development of strategic plan and guidelines | NTD-news | Neglected tropical diseases. [cited 4 Apr 2024]. Available: https://www.emro.who.int/neglected-tropical-diseases/ntd-news/news.html

23. World Health Organization. Ending the neglect to attain the Sustainable Development Goals: a road map for neglected tropical diseases 2021–2030. Geneva: World Health Organization. 2020. Available: https://apps.who.int/iris/handle/10665/341313

24. Burt TP, Weerasinghe KDN. Rainfall distributions in Sri Lanka in time and space: An analysis based on daily rainfall data. Climate. 2014;2: 242–263. doi:10.3390/cli2040242

25. Alahacoon N, Edirisinghe M. Spatial variability of rainfall trends in sri lanka from 1989 to 2019 as an indication of climate change. ISPRS Int J Geoinf. 2021;10: 84. doi:10.3390/ijgi10020084

26. Lane RP, Pile MM, Amerasinghe FP. Anthropophagy and aggregation behaviour of the sandfly Phlebotomus argentipes in Sri Lanka. Med Vet Entomol. 1990;4: 79–88. doi:10.1111/j.1365-2915.1990.tb00263.x

27. Hersbach H., Bell B., Berrisford P., Biavati G., Horányi A., Muñoz Sabater J., et al. ERA5 hourly data on single levels from 1940 to present. Copernicus Climate Change Service (C3S) Climate Data Store (CDS). [cited 9 Sep 2023]. doi:10.24381/cds.adbb2d47

28. Bolton D. The computation of equivalent potential temperature. Mon Weather Rev. 1980;108: 1046–1053. doi:10.1175/1520-0493(1980)108<1046:TCOEPT>2.0.CO;2

29. Survey Department of SriLanka. 2024 [cited 10 Mar 2024]. Available: https://www.survey.gov.lk/

30. Di Marco M, Ferrier S, Harwood TD, Hoskins AJ, Watson JEM. Wilderness areas halve the extinction risk of terrestrial biodiversity. Nature. 2019;573: 582–585. doi:10.1038/s41586-019-1567-7
31. Keys PW, Barnes EA, Carter NH. A machine-learning approach to human footprint index estimation with applications to sustainable development. Environ Res Lett. 2021;16: 044061. doi:10.1088/1748-9326/ABE00A
32. Gasparrinia A, Armstrong B, Kenward MG. Distributed lag non-linear models. Stat Med. 2010;29: 2224–2234. doi:10.1002/sim.3940
33. Gasparrini A, Armstrong B. Reducing and meta-analysing estimates from distributed lag non-linear models. BMC Med Res Methodol. 2013;13: 1–10. doi:10.1186/1471-2288-13-1
34. Chen T, Guestrin C. XGBoost: A scalable tree boosting system. Proceedings of the ACM SIGKDD International Conference on Knowledge Discovery and Data Mining. Association for Computing Machinery; 2016. pp. 785–794. doi:10.1145/2939672.2939785
35. Gasparrini A, Armstrong B, Kenward MG. Multivariate meta-analysis for non-linear and other multi-parameter associations. Stat Med. 2012;31: 3821–3839. doi:10.1002/sim.5471
36. Huedo-Medina TB, Sánchez-Meca J, Marín-Martínez F, Botella J. Assessing heterogeneity in meta-analysis: Q statistic or I 2 Index? Psychol Methods. 2006;11: 193–206. doi:10.1037/1082-989X.11.2.193
37. Bhaskaran K, Gasparrini A, Hajat S, Smeeth L, Armstrong B. Time series regression studies in environmental epidemiology. Int J Epidemiol. 2013;42: 1187–1195. doi:10.1093/ije/dyt092
38. Gasparrini A, Armstrong B, Kenward MG. Multivariate meta-analysis for non-linear and other multi-parameter associations. Stat Med. 2012;31: 3821–3839. doi:10.1002/sim.5471
39. Lundberg SM, Erion G, Chen H, DeGrave A, Prutkin JM, Nair B, et al. From local explanations to global understanding with explainable AI for trees. Nat Mach Intell. 2020;2: 56–67. doi:10.1038/s42256-019-0138-9
40. R Core Team. R: A language and environment for statistical computing. R

Foundation for Statistical Computing, Vienna, Austria. Vienna, Austria: Version 4.1.0 (2021-05-18); Available: https://www.r-project.org/

41. Laurent BS, Oy K, Miller B, Gasteiger EB, Lee E, Sovannaroth S, et al. Cow - baited tents are highly effective in sampling diverse Anopheles malaria vectors in Cambodia. Malar J. 2016;15: 1–11. doi:10.1186/s12936-016-1488-y

42. Gebresilassie A, Yared S, Aklilu E, Kirstein OD, Moncaz A, Tekie H, et al. Host choice of Phlebotomus orientalis (Diptera : Psychodidae) in animal baited experiments : a field study in Tahtay Adiyabo district , northern Ethiopia. Parasit Vectors. 2015;8: 1–10. doi:10.1186/s13071-015-0807-4

43. Surendran SN, Sarma DK, Jude PJ, Kemppainen P, Kanthakumaran N, Gajapathy K, et al. Molecular characterization and identification of members of the Anopheles subpictus complex in Sri Lanka. Malar J. 2013;12: 1–11. doi:10.1186/1475-2875-12-304

44. Gajapathy K, Tharmasegaram T, Eswaramohan T, Peries LBSL, Jayanetti R, Surendran SN. DNA barcoding of Sri Lankan phlebotomine sand flies using cytochrome c oxidase subunit I reveals the presence of cryptic species. Acta Trop. 2016;161: 1–7. doi:10.1016/j.actatropica.2016.05.001

45. Pathirage DRK, Weeraratne TC, Senanayake SC, Parakrama Karunaratne SHP, Karunaweera ND. Genetic diversity and population structure of Phlebotomus argentipes: Vector of Leishmania donovani in Sri Lanka. PLoS One. 2021;16: 1–13. doi:10.1371/journal.pone.0256819

46. Elnaiem DE, Khogali A, Alsharif B, Dakein O, Jibreel T, Hassan M, et al. Understanding sand fly sampling methods: Sticky traps are attraction-based and not interceptive sampling tools of Phlebotomus orientalis. Parasites and Vectors. 2020;13: 1–15. doi:10.1186/s13071-020-04249-1

47. Murray HW, Berman JD, Davies CR, Saravia NG. Advances in leishmaniasis. Lancet. 2005;366: 1561–1577. doi:10.1016/S0140-6736(05)67629-5

48. Claborn DM. The Biology and Control of Leishmaniasis Vectors. J Glob Infect Dis. 2010;2: 127. doi:10.4103/0974-777X.62866

49. Feliciangeli MD. Natural breeding places of phlebotomine sandflies. Med Vet Entomol. 2004;18: 71–80. doi:10.1111/j.0269-283X.2004.0487.x

50. Ganser C, Wisely SM. Patterns of spatio-temporal distribution, abundance, and diversity in a mosquito community from the eastern Smoky Hills of Kansas. J Vector Ecol. 2013;38: 229–236. doi:10.1111/j.1948-7134.2013.12035.x

Figure legends

Fig 1 Leishmaniasis annual average incidence rate.

Leishmaniasis annual average incidence rate 2018-2020 (per 1000 population) by districts and locations of sand fly surveillance sites in Sri Lanka. Black solid lines in the map represent the boundaries of administrative districts. Green shaded circles indicate the location of long-term sand fly surveillance sites. Source of the base file: https://data.humdata.org/dataset/sri-lanka-administrative-levels-0-4-boundaries.

Fig 2 Relative risk (RR) of leishmaniasis vector activity.

Relative risk (RR) of leishmaniasis vector activity (measured by UV LED CDC traps) by rainfall (a), ambient temperature (maximum temperature) (b), average relative humidity (c), sunshine hours (d), evaporation (e) and wind speed (f) at a lag of 0 months. The exposure-response functions at lag of 0 month were predicted from the pooled exposure-response function obtained from the meta-analysis for all surveillance sites in Sri Lanka, 2018–20. Shaded areas are 95% CIs. Relative risks were calculated with reference to the risk at a rainfall value of 0 mm per month, maximum temperature of 29.3^{0}C, average relative humidity of 72.25, average evaporation of 3.3mm and wind speed of kmh^{-1}. The most important lags for each exposure variable were selected for presentation. The full spectrum of exposure-lag response associations is given in the appendix (pp 7–14).

Fig 3 Relative risk (RR) of leishmaniasis vector activity by soil temperature.

Relative risk (RR) of leishmaniasis vector activity (measured by UV LED CDC traps) by soil temperature measured at 8.30 am at 10cm below the surface (panel a) and evaporation (panel b) at the lag of 2 months. The exposure-response functions at each lag were predicted from the pooled exposure-response function obtained from the meta-analysis for all surveillance sites in Sri Lanka, 2018–20. Shaded areas are 95% CIs. Relative risks were calculated with reference to the risk at a soil temperature of 26^0C. The full spectrum of the associations is given in supplementary figures (S13-S14)**.**

Click here to access/download
Supporting Information
Supplement_PLOS_15.04.2024.docx
