# Supplementary material for: Impact of climate and land use on the temporal variability of sand fly density in Sri Lanka: A 2-year longitudinal study
Source: PLoS Negl Trop Dis. 2024 Nov 21;18(11):e0012675. doi: 10.1371/journal.pntd.0012675 (PMC11620634; doi:10.1371/journal.pntd.0012675)
Supplement: Attachment — Submitted filename: $$2024e_REVIEW-to-PLOS.docx [file pntd.0012675.s002.docx]

PNTD-D-24-00582 PLOS Neglected Tropical Diseases

“Impact of climatic factors on the temporal variability of sand fly abundance in Sri Lanka: a 2-year longitudinal study.”

Senanayake, Sanath … Karunaweera, Nadira.

Improved modes of expression, misspellings, grammatical errors are listed below.

British English spellings were not commented upon, such as substituting “s” for the USA “z” and several other such.

Abstract: Total cases of CL per annum, total numbers of sand flies and other summary numerical data will be helpful to describe the scope of the problem and the scale of comparisons and the positive findings.

l.43 “We found that climate plays …” = Climate played …

l.50 “; later 2 variables with a 2 month time lag.” = , a 2-month lag period was associated with RR.

l.58 Delete “would”

l.60-61 Make a second sentence.

l.64 Place comma after “tropics”

l.65 “Thus, we aimed to understand” =The goal herein was to understand

l.69 “divers” = drivers; Delete “We found that c” = C; “major” = significant

l.75 “A high” = High

l.88 “Phlebotomine” – Phlebotominae

l.89 “hairy” = , heavily setose,

l.95 Delete “in large part”

l.100 Delete “It is estimated that b” = B

l.103 “over” = approximately? [“over” is editorializing]

l.108 Somewhere you must indicate that several other genera of Phlebotominae fit the description that follows; indicate that Phlebotomus genus is only found in the Afro-Asia-European regions.

l.110 “include” = consist of

l.116 Use tilde over the “n” in El Nino.

l.117 “different” = a variety of

l.118-119 “This heterogeneity could be largely due to..” = These inconsistencies undoubtedly are due to

l.125 Real numbers are necessary to demonstrate this “exponential rise” For example, ‘From 2000 to 2024, leish cases rose from 1,200 to the current 7,000 per annum.’

l.128 “compared to” Do you mean ‘in contrast to’ or ‘similar to’ … the mainland species.

1.140-141 Make into a separate sentence.

l.149 How much “over” is over? Too non-specific.

l.166 Delete “We also considered t” = T; “as” = was

l.168 “the township they belonged to” = the township in which they were located

l.217 delete “We further utilized t” = T …data were downloaded …

l 230 “, which could further” = that can

l.240 delete “we utilized”

l.241 “, which integrates’ = integrated

l.248 delete “We utilized the most updated” = Updated

l.279 “association” = associations [to fit with the “were” that follows]

l.296 “We used lag up” = A lag of up

l.297	“considering” = considered

l.307 Is it “sandfly” or “sand fly”? Typically no space (sandfly), but the draft uses this inconsistently.

l.309 Do you mean ‘ a better model fit for the monthly total of LT compared with that of the CBNT for all weather parameters’?

l.310 “we selected” = the; “our” = the; “obtained using” = from the’; “for our” = were used for [Is this correct? Your sentence was a little confusing.

l.313 “We used” = The; “for” = provided

l.319 Delete “, that”; “boosting, has” = boosting which has

l.325 “allows us to rank” = allowed ranking

l.331 “identified” = were identified; end the sentence at Table 1. {Sentence too long, reorganize.}

The following descriptions of the analysis using the first person plural is distracting and perhaps can be better understood in the form of a table.

L354 Begin sentence with full word (not abbreviation) = *Phlebotomus.*

l.358 “there” = males; delete the later ‘males’

Table 2:

Data are easier to read if the ‘thousands’ are delimited by a comma, e.g., “4619” = 4,619

In the legend, spell out the first “P.” Note that “different” == two; define your abbreviations in the legend as well – LT = CDC light trap, CBNT = cattle-baited tent traps

l.376 Delete “in these areas” (unnecessary)

l.377 Stop sentence after (Fig 1). “though” = However,

Fig.1

Nice figure! On it change “Incidence rate by District” to *Leishmania* case rate by District

L.392-396 Break this flux of information into at least 3 readable sentences.

l.570	Add a note that flies product a nearly equal sex ratio in colony. Seems that female flies are on cow as well; the explanation for the difference is very tenuous.

l.576 Listing and explanation of a few more of these variables is of interest, particularly since this comparison is the main point of this study.

l.625 “In our study we observed that”= The current study also indicated that

l.637 “utilzed” = useful

l.659 “generalizability” = ??

l.667 add a comma after “study”

l.843 “sri lanka” = capitalized?
