# Supplementary material for: Impact of climate and land use on the temporal variability of sand fly density in Sri Lanka: A 2-year longitudinal study
Source: PLoS Negl Trop Dis. 2024 Nov 21;18(11):e0012675. doi: 10.1371/journal.pntd.0012675 (PMC11620634; doi:10.1371/journal.pntd.0012675)
Supplement: Attachment — Submitted filename: Response_to_reviewers.docx [file pntd.0012675.s004.docx]

**Impact of climate and land use on the temporal variability of sand fly abundance in Sri Lanka: A 2-year longitudinal study**

**Response to reviewers**

**Reviewer 1**

**R1.1:** The statistical analysis was impressive and can serve as model for analyses of ecological risk data. The choice of sampling methods and sites were reasonable and well described.

Thank you for your encouraging comment and taking time to review our manuscript.

**R1.2:** Abstract: Total cases of CL per annum, total numbers of sand flies and other summary numerical data will be helpful to describe the scope of the problem and the scale of comparisons and the positive findings.

Thank you for highlining this important consideration. We have updated the abstract and included following information.

The reported leishmaniasis cases were increasing nearly three folds over past decade, from 1,367 in 2014 to 3714 in 2023, as per records maintained by the Epidemiology Unit, Ministry of Health, Sri Lanka (<https://www.epid.gov.lk/home>). Please see line 35 to 37.

The total number of *P.argentipes* captured over the study period was 36,648. Please see line 42.

**R1.3:** The limitations of placement of traps might have been discussed, but the large number of traps per location will have compensated.

Thanks for highlighting this important point, which is now addressed under ‘limitations. Line 682 to 688.

**R1.4:** Descriptions of the many levels of statistical analysis became difficult to follow, however. A table or even a flow chart can clarify name of program, data analyzed, question answered.

Thank you for your valuable feedback. We agree with your comment and have simplified the description of the statistical analysis. Some details have been moved to the supplementary material (please see sections 3, 4 5 and 6 in the supplement). The analytical steps are now summarized in a box in the supplementary appendix (S1, Box 1) and referenced in the main text (Lines 282–283).

**R1.5:** Excellent maps and clear graphs contribute to visualizing Sri Lanka, trap placement and fly data. Use of commas to indicate 'thousands' is recommended. Plan = analysis.

Thank you for the comment. We have now modified the map using commas to indicate thousands. The figure label change to sand fly density to be compatible with the text.

**R1.6:** Graphic indication of effect of each variable on RR was quite interesting.

Thank you for the encouraging comment.

**R1.7:** A table of ranges for each of the 10 sites will help to indicate the degree to which trap placement influences sand fly attraction to the trap (SD perhaps).

Thank you for your insightful comment. Unfortunately, individual trap data was not recorded due to logistical reasons. The sand fly collections in each site at each collection time were pooled, and sand flies were counted and identified. As a result, we are unable to provide trap-specific ranges. However, we presented site-specific data summaries in the supplementary material. The S1 Fig. 4 illustrate the temporal and spatial variability of sand fly counts by sentinel site over the 24-month study period across all study settings. The interquartile range (IQR) of each boxplot (panel A and B) represents the middle 50% of the data, while the whiskers extend to show the overall variability. The line diagrams (panel C and D) depict the average sand fly counts by month across all sentinel sites, highlighting seasonal trends and temporal fluctuations. The S1 Table 1 provides summary statistics of sand fly counts, including minimum, maximum, mean, median, and standard deviation, to reflect the variability across individual months for each sentinel site.

**R1.8**: Conclusions supported by data. Limitations of data were addressed to some extent. Undoubtedly, very large trap-to-trap variation existed within each of the 10 sites, this should have been expressed in a table of ranges to indicate that trap placement and very local environment influences sand fly attraction, and hence inferences of density.

Thank you for your comment. As explained above, within a site, trap to trap analysis was not possible. However, the environmental influence on sand fly density figures between sites are shown in supplementary plot Fig 4, panel A and B. We have further discussed this in the discussion highlighting the importance of novel surveillance methods based on AI technologies (smart traps). Table indicating the summary statistics of CBNT and light trap counts by sentinel site averaged across all the months has been added to the supplement. Please see the Table 1 of the additional material. This information has been included in the main text under the sub heading “spatial and temporal patterns” line 380 to 387.

**R1.9:** The life cycle of the fly was indicated in the introduction; but the potential effect of each of the climatic variables was not indicated. For example, because eggs and larvae develop in the soil (evidence for depth from literature?), the soil temps are important--as was finally noted in the conclusions.

Thank you for the comment. Information highlighting the potential influence of different climate variable has been included with following references cited (Reference 60 and 64).^[1],^^[2]^

**R1.10:** What's the significance of wind -- e.g., adults cannot fly well.

Thank you for the comment. While sand flies may not be strong fliers, low wind speed plays a crucial role in their ability to move, locate hosts, and survive in their environments.^[3],^^[4]^ Research indicates that sandflies are highly sensitive to wind speed and tend to favor breeding sites that offer shelter from the wind.^[5]^ Moreover, elevated wind speeds can significantly reduce or even completely inhibit their flight activity.^[6]^

**R1.11:** The unbalanced sex ratio in light traps seems to indicate a serious sampling error. Detailed explanations are expected.

Thank you for this comment. We have modified the Table 2 of the main text to include the number and ration of male and female sand flies by each sentinel site (line 377). The sex ratio of all the sites showed male dominance. The unbalanced sex ratio in CBNTs has been well described in the previous literature,^[7],^^[8]^ which has been added to the discussion with the respective references (line 595-601).

**R1.12:** Editorial and data presentation/modifications

*l.43 “We found that climate plays …” = Climate played …*

The change has been applied. Please see line 46

*l.50 “; later 2 variables with a 2 month time lag.” = , a 2-month lag period was associated with RR.*

We modified the sentence following to the comments made by other reviewers. Please see lines 49 to 52.

*l.58 Delete “would”*

Deleted.

*l.60-61 Make a second sentence.*

The sentence has been modified

*l.64 Place comma after “tropics”*

The sentence has been modified as per the comments made by other reviewers (line 65).

*l.65 “Thus, we aimed to understand” =The goal herein was to understand*

The suggested phrase has been introduced.

*l.69 “divers” = drivers; Delete “We found that c” = C; “major” = significant*

Suggested changes have been applied.

*l.75 “A high” = High*

The sentence changed as per the comments made by other reviewers. “. Large land areas of chena and coconut plantations, along with sparse forests, homesteads, and reduced human footprint indices, positively influenced sand fly abundance.” Please see line 77 to 79.

*l.88 “Phlebotomine” – Phlebotominae*

The change has been applied.

*l.89 “hairy” = , heavily setose,*

The replacement has been made.

*l.95 Delete “in large part”*

The change has been applied.

*l.100 Delete “It is estimated that b” = B*

The change has been applied.

*l.103 “over” = approximately? [“over” is editorializing]*

“over” replaced by “approximately”

*l.108 Somewhere you must indicate that several other genera of Phlebotominae fit the description that follows; indicate that Phlebotomus genus is only found in the Afro-Asia-European regions.*

This information was added to the introduction. Please see line 89 – 90.

*l.110 “include” = consist of*

The change has been applied.

*l.116 Use tilde over the “n” in El Nino.*

The change has been applied.

*l.117 “different” = a variety of*

The change has been applied .

*l.118-119 “This heterogeneity could be largely due to..” = These inconsistencies undoubtedly are due to*

The preceding sentence changed as per the comments made by other reviewers. The heterogeneity is now replaced by diversity to be consistent with the previous change (line 122 to 124).

*l.125 Real numbers are necessary to demonstrate this “exponential rise” For example, ‘From 2000 to 2024, leish cases rose from 1,200 to the current 7,000 per annum.’*

Thank you for the comment. The information is now included. Please see line 129 to 131.

*l.128 “compared to” Do you mean ‘in contrast to’ or ‘similar to’ … the mainland species.*

Thank you. ‘in contrast to’ was introduced (line 133).

*1.140-141 Make into a separate sentence.*

The change applied (line 142 to 146).

*l.149 How much “over” is over? Too non-specific.*

The annual average rainfall is given (line 155 to 156).

*l.166 Delete “We also considered t” = T; “as” = was*

The change has been applied and the sentence modified (line 171 to 176).

*l.168 “the township they belonged to” = the township in which they were located*

The change has been applied (line 176).

*l.217 delete “We further utilized t” = T …data were downloaded …*

The sentence was changed per the comments of other reviewers. “We supplemented the ground-level weather data using the ERA5-Land reanalysis climate dataset, accessible through the Copernicus Climate Change Service Climate Data Store.” Please see line 224 to 228.

*l 230 “, which could further” = that can*

The change has been applied.

*l.240 delete “we utilized”*

The sentence has been changed as per the comments of other reviewers.

*l.241 “, which integrates’ = integrated*

The sentence has been changed as per the comments of other reviewers (line 243 to 245).

*l.248 delete “We utilized the most updated” = Updated*

The change has been applied, and the sentence has been modified (line 249).

*l.279 “association” = associations [to fit with the “were” that follows]*

The change has been applied (line 281).

*l.296 “We used lag up” = A lag of up*

Thank you. The sentence has been revised (line 303 to 308).

*l.297	“considering” = considered*

The sentence has been changed. “A lag of up to three months was considered to account for the life cycle of sand fly vectors, ensuring that all biologically plausible associations between climate variables and sand fly densities were captured.” Please see line 303 to 308.

*l.307 Is it “sandfly” or “sand fly”? Typically no space (sandfly), but the draft uses this inconsistently.*

Thank you for the comment. Sand fly has been used consistently throughout the manuscript.

*l.309 Do you mean ‘ a better model fit for the monthly total of LT compared with that of the CBNT for all weather parameters’?*

We used three sandfly indices as the outcome variables (LT per trap, LT total and CBNT per trap) The model fit of LT average was better compared to both CBNT and LT monthly totals. Please also see the Table 3 of additional material. The text in the manuscript has been changed to improve clarity (line 317 to 321).

*l.310 “we selected” = the; “our” = the; “obtained using” = from the’; “for our” = were used for [Is this correct? Your sentence was a little confusing.*

Thank you for the comment. We modified the sentence to improve the clarity.

“Therefore, we selected sand fly densities obtained using the LT per trap for the primary analysis and compared the results with that of CBNT per trap where relevant.” Please see the revised text in line 331 to 323

*l.313 “We used” = The; “for” = provided*

The sentence has been changed.

*l.319 Delete “, that”; “boosting, has” = boosting which has*

The change has been applied (line 329).

l.*325 “allows us to rank” = allowed ranking*

The change has been applied (line 335).

*l.331 “identified” = were identified; end the sentence at Table 1. {Sentence too long, reorganize.}*

The sentence has been changed. Thank you for the comment.

“All lagged climate variables, identified using the DLNM approach described above, were incorporated into the XGBoost model, along with the non-climate variables listed in S1 Table 1.” Please see line 341 to 344.

*The following descriptions of the analysis using the first-person plural is distracting and perhaps can be better understood in the form of a table.*

Thank you for your feedback. To enhance the clarity of our overall statistical approach, we have included a high-level summary of the methodology in the supplement (S1 Box 1). Additionally, a detailed, step-by-step description of the machine learning approach is provided in S1, Table 6.

*L354 Begin sentence with full word (not abbreviation) = Phlebotomus.*

The change has been applied (line 354).

*l.358 “there” = males; delete the later ‘males’*

The sentence was rephrased to improve clarity.

*Table 2:*

*Data are easier to read if the ‘thousands’ are delimited by a comma, e.g., “4619” = 4,619*

*In the legend, spell out the first “P.” Note that “different” == two; define your abbreviations in the legend as well – LT = CDC light trap, CBNT = cattle-baited tent traps*

The requested changes have been applied to the table 2.

*l.376 Delete “in these areas” (unnecessary)*

The change has been applied

*l.377 Stop sentence after (Fig 1). “though” = However,*

The change has been applied.

*Fig.1*

*Nice figure! On it change “Incidence rate by District” to Leishmania case rate by District*

Thank you for your comment. We have changed the text to adhere with the proper terminology. Annual leishmaniasis incidence by district per 100,000 population.

*L.392-396 Break this flux of information into at least 3 readable sentences.*

Thank you for the information. The section has now been modified (line 413 to 417).

*l.570	Add a note that flies product a nearly equal sex ratio in colony. Seems that female flies are on cow as well; the explanation for the difference is very tenuous.*

Thank you for the comment. Relevant information has been cited, which supports male dominance in sand fly trap collections (line 597-599).

*l.576 Listing and explanation of a few more of these variables is of interest, particularly since this comparison is the main point of this study.*

Thank you for the comment. We have revised the discussion including the information suggested in several instances.

*l.625 “In our study we observed that”= The current study also indicated that*

The change has been applied.

*l.637 “utilzed” = useful*

The change has been applied.

l.659 “generalizability” = ??

The sentence was rephrased. A new section is added.

*l.667 add a comma after “study”*

The change has been applied. Please see line 728.

l.843 “sri lanka” = capitalized?

The references revised.

**Reviewer 2**

Major Issues

**R2.1** The authors mention in the limitations, that probably a generalization (which the authors tend to assume) cannot be done. There are a more than a few variables associated with heterogeneity. This is in line with the heterogeneous climate in Sri Lanka: “four climatic zones based predominantly on the rainfall, viz. wet zone, intermediate zone, dry zone, and semi-arid zone”. Shouldn’t you have in account these different zones when you look for associations between “between weather variables? Can you do the same exercise for each of the different climatic zones?

Thank you for pointing out this major concern. We agree that the heterogeneity across the study settings for the said weather variables can be partly explained as effect modification by the climate zones they belong to. To overcome the uneven distribution of the study settings in climate zones and for the ease of interpretation of estimates across all zones, we used the same two-staged hieratical metanalytical approach previously described in the methods section. This present approach allowed us to account for and explore the impact of climate zones—or their moderator effect—on the associations between weather variables and sand fly abundance. For this sub-analysis, we selected weather variables that exhibited statistically significant heterogeneity. We used the annual average rainfall of each study site as the meta-predictor in the second-stage meta-analysis to predict the relative risk across the range of annual average rainfall values in our sample, representing the three climate zones. Our analysis focused on the wet, intermediate, and dry zones, as there were no study sites in the semi-arid zone. Please see the revised methods section (line 559 to 570) where we include this analysis. We have included a section to the supplement (S1 Section 5) providing methodological details and the results. Please see S1 Table 6 and S1 Fig 14 under S1 Section 5 (Moderator effects of climate zones on weather-sand fly association).

We observed that the relative risk of the association between weather and sand fly abundance increased as the climate zones shifted from wet to dry (S1 Fig. 14). This suggests a positive moderator effect of the dry zone on the exposure-response relationship compared to the wet zone. However, these moderator effects were not statistically significant, as indicated by the Wald test, and the Q-test of heterogeneity remained statistically significant even after accounting for climate zones. A new section has been incorporated into the results to present the findings (lines 559 to 570), which are further expanded in the discussion section (lines 655 to 660).

This finding further supports our approach of incorporating non-climate variables within a broader geographical context (5 km radius) to characterize the ecological features of the climate zones around the surveillance sites and explore their relative contribution to sand fly abundance. Please refer to the revised methods section and supplementary information for a detailed account of the impact of climate zones on each weather variable considered.

**R2.2:** While reading the results, I was under the impression you only used the UV LED CDC trap-derived sandfly density in the association analysis (e.g. “When pooled across all the sentinel sites, the rainfall appeared to be associated with the risk of increasing sand fly density measured by UV LED CDC trap at lag of 0”). Why did you exclude the collection data obtained in the context of CBNTs, via which the majority of sandflies were collected in this study?

Thank you for your comment. As mentioned in the methods section, we did include sand fly abundance data collected using CBNTs in our analysis. We have modified the text to improve the clarity and avoid confusion (line 298 to 300). However, the CBNT data showed a poor model fit with high AIC values, and the results were not statistically significant (line 310 to 314 in the manuscript and Table 3 in supplement). The results of this analysis was not statistically significant and the direction of the association was consistent with the UV LED CDC trap-derived data, and no additional insights were gained. To avoid unnecessary complexity, we did not include these results in the main manuscript. For completeness, we have now added a figure in the supplementary materials showing the exposure-lag-response relationship between weather and sand fly abundance measured by CBNTs (S1 Fig 13). We have also discussed these findings and their comparison in the discussion section at lines 604 to 609.

**R2.3:** It would be interesting to understand whether, and to what extent, the different climatic parameters correlate(e.g. rainfall and humidity, temperature and humidity…). Can you do that kind of analysis?

Thank you for your comments. We have included a correlation matrix demonstrating the magnitude, direction and statistical significance of the correlation between climate variables. The results are presented in the supplementary section under exploratory data analysis (Fig. 5) and the reference to this has been made in the manuscript (line 234 to 235).

**R2.4:** The collection methods need further details. Did you collect in arbitrary days regardless of the weather? Did you collect always at the same time? Thinking on “daylight hours”, did you always collect when it was dark?

As described in the methods section, a net trap with a cow inside was used for each collection. The collection time was kept constant as much as possible, for all sites in every visit. Each site was visited approximately on the same day of the month. Day time collections were not done based on previous experience and literature.^[9]^

CDC light traps. The traps were kept outdoors and hung on the roof of the pavement, surrounding the house. The 20 houses and the trap placement were constant throughout the study period.

**R2.4:** As per the non-climatic variables, I have some doubts on whether you can really measure a real impact, since I am assuming the collection was made under equal conditions (one ecotope selected and used throughout study sites) and you are considering a fairly large 5 Km perimeter. Was this choice based on previous similar studies? The discussion could be complemented with comparisons with other similar studies (in different geographies).

Thank you for your observation. We have modified the term “non-climate variables” to land use variables throughout to improve the clarity and precisely mention what we measured. The choice of incorporating non-climatic (land use) variables within a 5 km radius was informed by several factors, including the need to capture a comprehensive ecological context that could influence sand fly abundance beyond immediate microhabitats. Although we aimed to standardize collection conditions by selecting similar ecotopes across study sites, sand fly populations are influenced by a variety of environmental factors that operate at different spatial scales, including land use, vegetation cover, and human activities, which can vary even within seemingly similar ecotopes.

The 5 km radius was chosen after a careful ground level and remote (using ArcGIS) observations made in consultation with geographic experts to balance capturing relevant environmental variability while avoiding excessive spatial dilution of effects. This scale has been supported by previous studies that have shown that sand fly populations can be influenced by landscape features and environmental conditions extending several kilometers from the collection site.^[10]^ However, we acknowledge that this choice involves a trade-off: while it allows us to consider broader ecological factors, it may also introduce variability that can obscure more localized influences. This methodology was chosen to ensure a comprehensive analysis while remaining consistent with established research practices in the field. This information is now included in the ‘Methods’ (lines 240 to 246) and discussed in the discussion section providing references (lines 666 to 671).

Please also refer to our previous response under your first concern.

**R2.5:** Additionally, you could also try discussing why there is a huge difference in the sex ration when you use different collection methods.

Thak you for your comment. We have updated the Table 2 to demonstrate the female to male sax ratio for all the sentinel sites to better illustrate our findings.

The results of the current study on the population count of male and female *P. argentipes* are consistent with previous research conducted by Dinesh et al. (2001)^[11]^, Addy et al. (1983), and Lane, Pile and Amerasinghe (1990)^[12]^. Additionally, our observation is consistent with all study settings.

Dinesh et al. (2001) reported a significantly higher number of male *P. argentipes* compared to females, with a female-to-male ratio of 1:8. Similarly, Addy et al. (1983) also noted a higher count of male *P. argentipes*. However, Lane, Pile and Amerasinghe (1990) found that male sandflies outnumbered females on cows by 19:1. This study was carried out in Kandy, Sri Lanka.

We have included this information in the discussion sections of the manuscript (595 to 601). Though a large number of males are seen in CBNT collections with a ratio of approximately 10:1 (male: female) there is no significance difference in male: female ratio when they are emerging from laboratory colonies (Lawyer *et al* 2017).^[13]^

Minor Points:

**R2.6:** Line 43: Please end the sentence with a final mark.

Thank you for pointing out this typo. The full stop was added at the end of the sentence.

**R2.7:** Line 46-48: There is something wrong in this sentence. You mention 3 RR values but only 2 variables.

Thank you for your comment. We have revised the sentence as follows (line 49 to 52).

“The maximum relative risk (RR) observed was 3.76 (95% CI: 1.58-8.96) for rainfall at 120 mm/month, 2.14 (95% CI: 1.04-4.38) for relative humidity at 82% (both at real-time). The maximum RR was 2.81 (95% CI: 1.09-7.35) for the ambient temperature at 34.5°C, and 11.6 (95% CI: 4.38-30.76) for soil temperature (both at a 2-month lag).”

**R2.8:** Line 108: why are you restricting to the genus Phlebotomus?

The focus is on *Phlebotomus* since that is the only genus recognized as a leishmaniasis vector in the Asian region (where *Lutzomyia* is absent). We have included this information to the introduction (line 94 to 95).

**R2.9:** Table 1: You did not study any semi-arid zone? If yes, please mention it in the table.

Thank you for your comment. We did not have a sample site in the semi-arid zone.

Accordingly, we have modified the abstract line 42 replacing “all” with “three main” climate zones.

**R2.10:** Table 1: Please indicate the actual incidence of CL in each of the provinces studied (instead of only yes/rare).

Thank you for the comment. We have modified the Table 1 to contain the CL incidence by district.

**R2.11:** Line 195: Were the light traps placed inside the houses?

The light traps were placed outdoors (hung on the roof of the pavement, surrounding the house). The description was added to the text Lines 194, 199 and 201.

**R2.12:** *Line 239: What do you mean with “reservoirs”?*

Thank you for the comment. We now changed the “reservoirs” to “water reservoirs (tanks)”.

**R2.13:** *Table 2: Please include the sex ratio for each collection site and method (individual by district and by LT and CBNT.*

Thank you for your comments. We have modified Table 2 by including sex ratios individually by district and by trapping method.

**R2.14:** *Line 372: Do you have enough data to extrapolate that the Delft Island is a “high sandfly density zone”?*

There are only 4 data points. Therefore, Delft Island data was included for descriptive purposes only. Sand fly density in each site, including Delft island is shown in Table 2.

**Reviewer 3**

The manuscript "Impact of climatic factors on the temporal variability of sand fly abundance in Sri Lanka: A 2-year longitudinal study" is a valuable longitudinal ecological study of sand flies which adds information about specific patterns observed in Sri Lanka. The data and analyses seem sound, but some inconsistencies and minor errors needs to be resolved, as well as minor methodological clarifications. In the attached commented pdf comments about errors, missing information and missing additional key references are included. Please use "Sand Fly “consistently and also refer to Sand fly "abundance", not burden.

Thank you so much for taking the time to review our manuscript and providing insightful comments. We have replaced the word sandfly with sand fly and burden with density for consistency.

**R3.1:** Line 107 and 108: Additional recommended references were added (line 109).

**R3.2:** Line 118: The term “reported associations are inconsistent” was corrected to “reported associations are area specific”(line 122).

**R3.3:** Line 166: This needs more details, what is included in the index.

Details on the human footprint index presented in the methods to improve the clarity (line 173).

**R3.4:** Line 177: Table 1 This table is better suited to the supplementary online material.

Thank you for your comment.

Table 1 gives descriptive information essential for understanding the nature of the study. Therefore, the authors would prefer to retain it.

**R3.5:** Line 215: Did you minimize the distance? Be specific?

We initially selected the surveillance sites and then assigned weather data from the nearest meteorological station based on the GPS coordinates of both the surveillance sites and the meteorological monitoring stations. This selection process was done in consultation with the Department of Meteorology. We have revised the text to improve clarity (lines 223 to 225).

**R3.6:** Line 217: Why the hourly repository, if your data is monthly?

To supplement our ground data, we used ERA5-Land data, which is available at an hourly frequency by default (https://cds.climate.copernicus.eu/cdsapp#!/dataset/reanalysis-era5-land?tab=form). Furthermore, the original data provided by the Department of Meteorology was at a daily frequency, which we then aggregated into monthly values for our analysis. To align with the time series of the original ground data and assess the temporal correlation, we first aggregated the hourly ERA5-Land data into daily intervals. This approach allowed us to evaluate the temporal correlation at a higher granularity, making it more appropriate for validating and supplementing the ground data before aggregating it into a monthly frequency.

**R3.7:** Line 219: “Remotely sensed” changed to “ERA5-Land reanalysis” data set.

**R3.8:** Line 222: How was the ERA data processed? Using Copernicus or the Google Earth Engine?

Thank you for your comment. We used Copernicus Climate Change Service Climate Data Store. The data were downloaded in netCDF file format and processed using R package ncdf4. We have updated the section in the revised manuscript to include this information for clarity (line 228 to 230).

**R3.9:** Line 226: “Month-specific variability” changed to “seasonality”

**R3.10:** Line 247: Use the same legends in the y axis. Currently this is difficult to read.

Thank you for the comment. We have now revised the legend in the Y-axis of Figure 4.

**R3.11:** Line 267: “ Secondly, we employed XGBoost, an ensemble decision tree method” changed to “ Secondly, we employed XGBoost, a boosted ensemble decision tree method”

**R3.11:** Line 342: How many times was this cross validation done?

This cross-validation process was repeated up to 10 times to reduce variability between splits and ensure robust and reliable performance estimates. We have included this information in the supplementary material (Section 6: XGBoost model building and validation) to simplify the methods section in response to feedback from other reviewers.

**R3.11:** Line 545: The discussion needs to better reflect the literature. Designs such as the presented could be used to evaluate control interventions. Also strengthen the discussion referring to classical studies about environmental factors and sand fly ecology.

Thank you for your comment and the suggested references. We have thoroughly revised the discussion section to better align with the existing literature and have incorporated the recommended references, which are now appropriately cited.

**R3.13:** Same as reference 4.

The repeated references were corrected.

1. Rutledge, L.C., Ellenwood, D.A., 1975. Production of Phlebotomine Sandflies on the Open Forest Floor in Panama: Hydrologic and Physiographic Relations. Environmental Entomology 4, 78-82.
2. Rutledge, L.C., Mosser, H.L., 1972. Biology of Immature Sandflies (Diptera: Psychodidae) at the Bases of Trees in Panama. Environmental Entomology 1, 300-309.
3. Fischer D, Moeller P, Thomas SM, Naucke TJ, Beierkuhnlein C. Combining Climatic Projections and Dispersal Ability: A Method for Estimating the Responses of Sandfly Vector Species to Climate Change. *PLoS Negl Trop Dis* 2011; **5**: e1407
4. Colacicco-Mayhugh MG, Grieco JP, Putnam JL, Burkett DA, Coleman RE. Impact of Phlebotomine Sand Flies on United States Military Operations at Tallil Air Base, Iraq: 5.
5. Killick-Kendrick R, Wilkes TJ, Bailly M, Bailly I, Righton LA. Preliminary field observations on the flight speed of a phlebotomine sandfly. *Trans R Soc Trop Med Hyg* 1986; 80: 138–42.
6. Orshan L, Szekely D, Khalfa Z, Bitton S. Distribution and Seasonality of Phlebotomus Sand Flies in Cutaneous Leishmaniasis Foci, Judean Desert, Israel. *J Med Entomol* 2010; 47: 319–28.
7. Lane RP, Pile MM, Amerasinghe FP. Anthropophagy and aggregation behaviour of the sandfly Phlebotomus argentipes in Sri Lanka. Med Vet Entomol. 1990 Jan;4(1):79-88. doi: 10.1111/j.1365-2915.1990.tb00263.x. PMID: 2132972.
8. Moncaz A, Gebresilassie A, Kirstein O, Faiman R, Gebre-Michael T, Hailu A, Warburg A. Attraction of phlebotomine sand flies to baited and non-baited horizontal surfaces. Acta Trop. 2013 Jun;126(3):205-10. doi: 10.1016/j.actatropica.2013.02.024. Epub 2013 Mar 13. PMID: 23499863.
9. Killick-Kendrick R. The biology and control of phlebotomine sand flies. Clin Dermatol. 1999 May-Jun;17(3):279-89. doi: 10.1016/s0738-081x(99)00046-2. PMID: 10384867
10. Fischer D, Moeller P, Thomas SM, Naucke TJ, Beierkuhnlein C. Combining Climatic Projections and Dispersal Ability: A Method for Estimating the Responses of Sandfly Vector Species to Climate Change. *PLoS Negl Trop Dis* 2011; **5**: e1407.
11. Dinesh DS, Ranjan A, Palit A, Kishore K, Kar SK. Seasonal and nocturnal landing/biting behaviour of Phlebotomus argentipes (Diptera: Psychodidae). *Ann Trop Med Parasitol* 2001; **95**: 197–202
12. LANE RP, PILE MM, AMERASINGHE FP. Anthropophagy and aggregation behaviour of the sandfly *Phlebotomus argentipes* in Sri Lanka. *Med Vet Entomol* 1990; **4**: 79–88.
13. Lawyer P, Killick-Kendrick M, Rowland T, Rowton E, Volf P. Laboratory colonization and mass rearing of phlebotomine sand flies (Diptera, Psychodidae). *Parasite* 2017; **24**. DOI:10.1051/PARASITE/2017041.
